# Disruption of orbitofrontal-hypothalamic projections in a murine ALS model and in human patients

**DOI:** 10.1186/s40035-021-00241-6

**Published:** 2021-05-31

**Authors:** David Bayer, Stefano Antonucci, Hans-Peter Müller, Rami Saad, Luc Dupuis, Volker Rasche, Tobias M. Böckers, Albert C. Ludolph, Jan Kassubek, Francesco Roselli

**Affiliations:** 1 Department of Neurology, Ulm University, Ulm, Germany; 2 CEMMA (Cellular and Molecular Mechanisms in Aging) Research Training Group, Ulm, Germany; 3 University of Strasbourg, Strasbourg, France; 4 Department of Internal Medicine II, Ulm University Medical Centre, Ulm, Germany; 5 Institute of Anatomy and Cell Biology, Ulm University, Ulm, Germany; 6 German Center for Neurodegenerative Diseases-DZNE, Ulm, Germany

**Keywords:** rAAV2, Agranular insula, Orbitofrontal cortex, Lateral hypothalamus, Hypermetabolism, Amyotrophic lateral sclerosis

## Abstract

**Background:**

Increased catabolism has recently been recognized as a clinical manifestation of amyotrophic lateral sclerosis (ALS). The hypothalamic systems have been shown to be involved in the metabolic dysfunction in ALS, but the exact extent of hypothalamic circuit alterations in ALS is yet to be determined. Here we explored the integrity of large-scale cortico-hypothalamic circuits involved in energy homeostasis in murine models and in ALS patients.

**Methods:**

The rAAV2-based large-scale projection mapping and image analysis pipeline based on *Wholebrain* and *Ilastik* software suites were used to identify and quantify projections from the forebrain to the lateral hypothalamus in the SOD1(G93A) ALS mouse model (hypermetabolic) and the Fus^ΔNLS^ ALS mouse model (normo-metabolic). 3 T diffusion tensor imaging (DTI)-magnetic resonance imaging (MRI) was performed on 83 ALS and 65 control cases to investigate cortical projections to the lateral hypothalamus (LHA) in ALS.

**Results:**

Symptomatic SOD1(G93A) mice displayed an expansion of projections from agranular insula, ventrolateral orbitofrontal and secondary motor cortex to the LHA. These findings were reproduced in an independent cohort by using a different analytic approach. In contrast, in the Fus^ΔNLS^ ALS mouse model hypothalamic inputs from insula and orbitofrontal cortex were maintained while the projections from motor cortex were lost. The DTI-MRI data confirmed the disruption of the orbitofrontal-hypothalamic tract in ALS patients.

**Conclusion:**

This study provides converging murine and human data demonstrating the selective structural disruption of hypothalamic inputs in ALS as a promising factor contributing to the origin of the hypermetabolic phenotype.

**Supplementary Information:**

The online version contains supplementary material available at 10.1186/s40035-021-00241-6.

## Background

Amyotrophic lateral sclerosis (ALS) is traditionally conceptualized as a neurodegenerative condition primarily affecting the upper motoneurons located in the primary motor cortex, and lower motoneurons located in the spinal cord, whose dysfunction and loss result in a relentless, progressive and ultimately fatal motor impairment [1]. More recently, hypermetabolism has been recognized as an important, non-motor clinical feature of ALS [2]. Epidemiological surveys have revealed that ALS patients display increased catabolism [2, 3] and that a lower body-mass index (BMI) constitutes a risk factor for ALS [4]. Furthermore, reduced levels of metabolic rate proxies such as plasma lipids and body fat content are predictors of survival in ALS patients [5–7], that is, weight loss is strongly correlated with shorter survival [8]. The increased catabolism may be a result of intrinsic hypermetabolism, as demonstrated in both ALS patients (in about 50–60% of cases [9–13]) and mutant SOD1 ALS mice [14–16]. Notably, metabolism and energy balance seem to be promising targets for intervention, since increasing caloric intake is beneficial for survival, particularly in fast-progressing ALS patients [17, 18].

However, currently there are limited studies on the ALS-related metabolic dysfunction. The hypothalamic system has recently been suggested to be involved in this metabolic dysfunction, as hypothalamic atrophy was found to correlate with the reduced BMI in ALS patients [19] as well as in pathological samples [20]. Besides the evidence of hypothalamic atrophy, the hypothalamic system may also participate in ALS in other ways. The hypothalamic nuclei receive and integrate inputs from a large fraction of the brain [21–24] in order to maintain proper balance of feeding, energy storage and energy expenditure [25]. Thus, disruption of these inputs may be sufficient to drive metabolic imbalances. In fact, the overall architecture of large-scale networks appears to be disturbed in ALS [26–30] and the ALS-related pathobiochemistry can affect a significant proportion of cortical and subcortical structures, in a pattern that evolves with disease progression [31, 32]. We have hypothesized that disruption and remodeling may occur with similar degrees in large-scale networks that provide inputs to the feeding-regulating lateral hypothalamic area (LHA), and disruption of this balance may be associated with the disruption of metabolism. In this study, we set out to identify selective disruptions of projections from the insular and orbitofrontal cortex to LHA in ALS mouse model as well as in ALS patients by using retrograde rAAV-2 tracing and magnetic resonance imaging (MRI)-diffusion tensor imaging (DTI) tracing.

## Methods

### Animals

All experimental procedures involving animals were performed in accordance with the guidelines for the welfare of experimental animals issued by the Federal Government of Germany; all experiments were approved by the Regierungspräsidium Tübingen under the animal license number 1390 and by the Ulm University Tierforschungszentrum Committee. No effort was spared to implement “3R” guidelines for animal experimentation.

The following strains from Jackson Laboratories were used in this study: B6SJL-Tg (SOD1*G93A)1Gur/J (Stock No: 002726; it has fast progression of disease due to multiple copies of the transgene, with 50% of survival at ~ 130 days of age [33]; henceforth mSOD), and B6.Cg-Gt (ROSA)26Sor^tm6/(CAG74ZsGreen)Hze/J^ (henceforth ZsGreen). To generate the mSOD/ZsGreen double transgenic mice, hemizygous mSOD1 males were crossed with homozygous ZsGreen^+/+^ females as previously reported (♂mSOD1^+^ x ♀ZsGreen^+/+^ [26]), due to the sterility of mSOD1 females, and the F1 progeny was used to retain a standardized mix of the two backgrounds (B6-SJL and B6-Cg)*.* The progeny included mSOD^+^/ZsGreen^+/−^ mice at the expected mendelian rate. mSOD^+^/ZsGreen^+/−^ is henceforth referred to as mSOD1 and mSOD^−^/ZsGreen^+/−^as WT.

The heterozygous B6.Fus^ΔNLS/+^ (henceforth kiFus) mice were provided by Luc Dupuis from the Faculté de Médecine, Strasbourg, France [34]. To generate the kiFus/ZsGreen double transgenic mice, heterozygous Fus males were crossed with homozygous ZsGreen^+/+^ females (♂kiFUS^+^ x ♀ZsGreen^+/+^) to maintain similarity with the mSOD1 breeding strategy, although kiFUS females are fertile. The progeny included kiFus^+^/ZsGreen^+/−^ mice at the expected mendelian rate. kiFus^+^/ZsGreen^+/−^ is henceforth referred to as kiFUS and kiFus^−^/ZsGreen^+/−^as WT.

The mice were group-housed at 2–5 animals per cage under a 12 h/12 h light/dark cycle with relative humidity between 40 and 60%, with free access to food and water. All mice expressing mSOD were routinely tested for motor impairment and euthanized in case of overt motor disability.

Since male and female mice differ substantially in progression rates of clinical and biological manifestation of motoneuron disease (e.g. [35]), the present study focused only on male mice.

The exploratory nature of this study prevented a formal calculation of the cohort size based on power calculations; we therefore estimated the group size (*n* = 3–6) based on the previously obtained dataset for motor cortex [26].

### Viral vectors

Retrograde rAAV2 [36] vectors encoding pmSyn1-EBFP-Cre (Addgene plasmid # 51507; kindly donated by Hongkui Zeng [37]) were obtained from Addgene  (Watertown, MA).

### Intracerebral injection

Intracerebral injection of rAAVs was performed as reported previously [26]. Briefly, 1 μl of viral suspension with a titer of 3 × 10^12^ viral genomes/ml and 1 μl of 1% Fast Green were freshly mixed and 300 nl of the mixture was loaded into a pulled-glass capillary. Pulling parameters were optimized to obtain a long and tapering capillary tip. The mice were pre-treated with buprenorphine (0.1 mg/kg) and meloxicam (1 mg/kg) 20 min before anesthesia with 5% sevoflurane/95% O_2_, and then positioned onto a stereotactic frame (David Kopf Instruments, Tujunga, CA) on a heated pad connected to a closed-loop system to maintain body temperature at 37 °C as monitored by a rectal probe. Continuous anesthesia was maintained with 3% sevoflurane/97% O_2_. Upon incision of the scalp, a burr hole was prepared using a hand-held microdrill at the LHA (coordinates AP = − 1.20 mm, ML = − 1.25 mm, DV = − 4.70 mm, according to Paxinos atlas, 2nd edition [38]), a position refined for the mouse strain under consideration of age.

Under visual inspection, the pulled glass capillary (tip closed) was inserted into the drilled hole and gently pushed down to verify that meninges could be penetrated without deflection of the capillary. Then the glass capillary was withdrawn and opened gently at the very tip using a pair of microscissors. Before lowering the glass capillary to the final position inside the brain, a thin layer of Dulbecco’s phosphate buffered saline (+Ca/+Mg) was applied to the skull, to generate a virus-free tip by capillary force during lowering of the glass capillary. Since the brain tissue may absorb some of the liquid out of the glass capillary through its own tissue/capillary force, the glass capillary was lowered at a speed no slower than 2 mm/s during further movement. The viral suspension was injected using a Picosprizer microfluidic device within 5 min, and the duration of one pulse was 10 ms. As the opening diameter of each capillary could be slightly different, the injection pressure was adapted between 20 psi and 60 psi to ensure a constant overall flow rate of 50–60 nl/min. After injection, the capillary was kept in place for 15 min and then withdrawn with a continuous movement (1 mm/s). The remaining pressure on the capillary was released before withdrawing it to prevent any residual virus suspension to be discharged into the capillary thread.

### Immunohistochemistry

The mSOD mice were sacrificed at postnatal day 40 (P40, injection at P25) or at P110 (injection at P95). The Fus mice were injected at P255 and sacrificed at P270.

For immunohistochemistry, the mice were trans-cardially perfused with PBS and then 4% PFA in PBS; after 18 h of post-fixation in 4% PFA, brains were cryoprotected in 30% sucrose/PBS, embedded in optimal cutting temperature compound and serially sectioned using a Leica CM1950 cryostat into 70-μm sections from AP + 2.6 mm to − 3.0 mm as previously reported [26].

Immunostaining for SOD1 (Anti SOD1, Sigma-Aldrich Chemie GmbH, Taufkirchen, Germany; HPA001401, 1:500), misfolded SOD1 (Misfolded SOD1 (B8H10), MédiMabs, Montréal, Canada; MM-0070-p, 1:250) and Fus (Anti Fus, Sigma-Aldrich Chemie GmbH, HPA008784, 1:300) was performed. Briefly, the sections were blocked in blocking solution (3% bovine serum albumin and 0.3% Triton in PBS) for 2 h at room temperature, and incubated with the primary antibody diluted in a blocking solution at 4 °C for 72 h. After 3 washes, they were incubated with the secondary antibody (Invitrogen, Carlsbad, CA; donkey anti-rabbit Alexa Fluor 568, #A10042; donkey anti-mouse Alexa Fluor 647, #A31571; or donkey anti-guinea pig 405) diluted at 1:500 in blocking solution for 2 h at room temperature in the dark. After three further washes in PBS, the sections were mounted onto microscopic glass slides with Gold Antifade Mountant (Invitrogen, #P36930).

### Image acquisition

The glass slides of serially-sectioned brains were first subject to visual assessment using a Leica DMIL equipped with a 2.5x/0.07 objective; brains that were injected at the wrong location or displayed obvious macroscopic artifacts were excluded at this stage and not processed further. The remaining brain sections were imaged using a slide-scanning microscope (Leica DMI6000B) equipped with a 5x/0.12 objective, with exposure time ranging from 300 ms (405 nm) to 800 ms (647 nm). All images were saved at the image size of 15,304 × 28,295 pixels with 16-bit depth. Images of the cortical areas immunostained for misfolded SOD1 were acquired using a Zeiss LSM710 confocal laser scanning microscope equipped with a 20x air objective. In total, 15 optical sections, each 1-μm thick, were acquired per region of interest (ROI).

### Neuron mapping

**Fig. 1:**
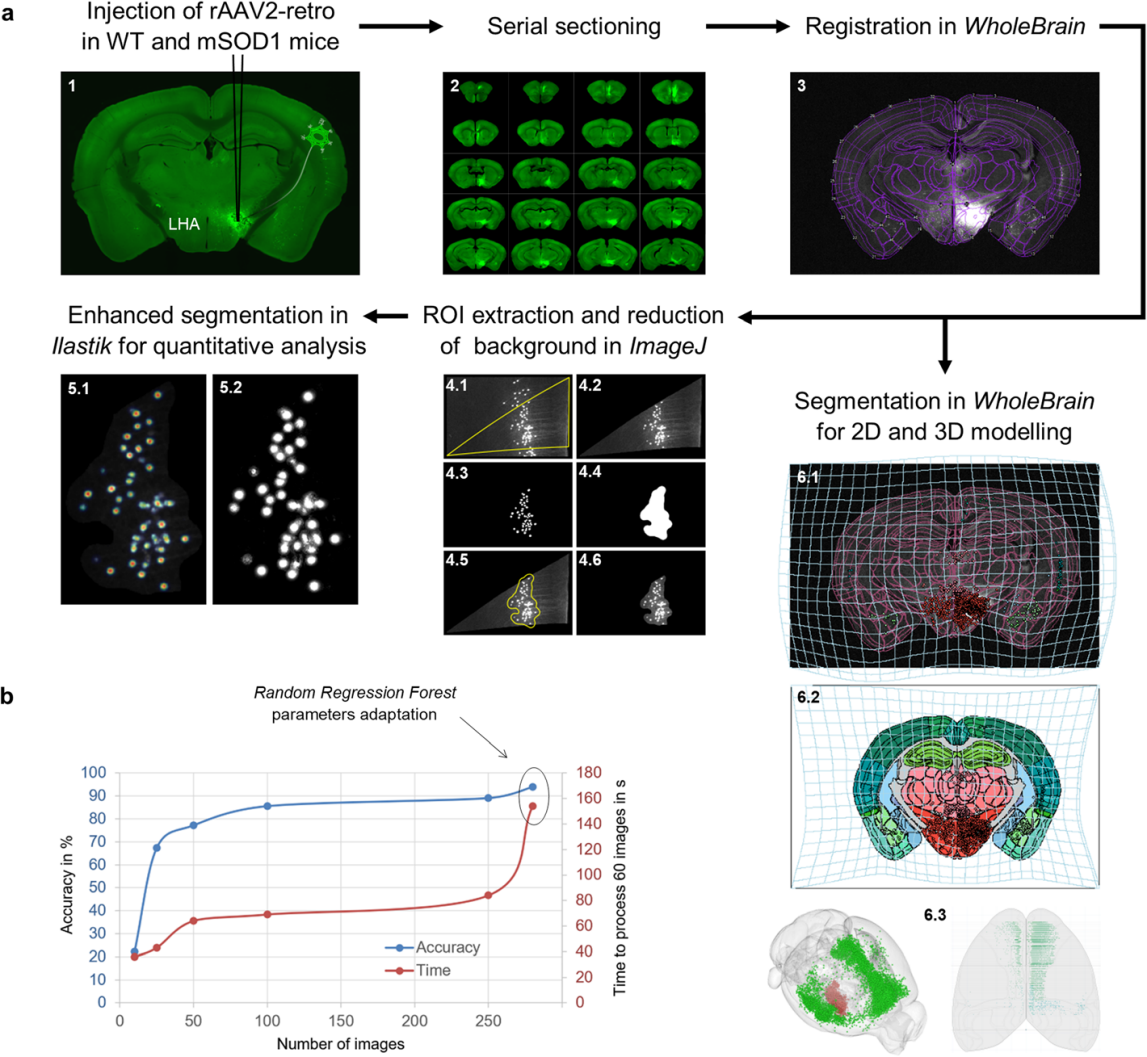
Pipeline for quantification of forebrain projections to the LHA. **a** Neurons projecting to the LHA were identified by injecting rAAV-retro encoding Cre into the LHA of WT (or mSOD1) carrying a floxed ZsGreen reporter allele (panel 1). Brains were serially sectioned (2) and each section was manually annotated in *WholeBrain* (3). For precise quantification of neurons in each anatomical structure, the registered sections were parcellated and refined in *Fiji* (panel 4), and then segmented using a purpose-trained *ilastik* project (Density Counting workflow) (5) to determine the final neuronal counts per image. The *WholeBrain* dataset was also used to derive an independent anatomical annotation in the Allen Brain Atlas (backward warp transform field [atlas plate to section], 6.1; forward warp transformation field [section to atlas plate] 6.2) and 3D reconstructions (6.3). **b** Training of the *ilastik* cell density counting suite. Accuracy improvement of the classifier according to the number of images used for training. The increasing need of computing power is indicated by the time needed to process 60 images. Accuracy was calculated by comparing randomly selected and manually analyzed images. A final accuracy of 94% was achieved with the use of 250 images after adaptation of parameters for the Random Regression Forest

A multiple-software approach was devised to analyze the images and properly parcellate and quantify projections from cortex to LHA (Fig. 1a).

Anatomical annotation **–** First, a custom *Fiji* [39] macro allowed to crop single brain sections out of whole microscopic slide images while appending serial numbers. Hands-on registration of coronal brain sections was performed by means of *WholeBrain* package in *R* [40], laying its foundations on the Allen Brain Mouse Reference Atlas (ABA, version 2011 [41]). For tilted sections (as compared to the original ABA coronal planes) and biological inter-individual brain differences, a series of easily recognizable anatomical landmarks were chosen to appropriately map the ROIs along the anterior-posterior axis (Table S1). The stereotactic coordinates were determined with *openbrainmap.org*.

Neuron segmentation **–** Despite the flexibility of the parameters of the built-in segmentation functions in *WholeBrain*, a systematic underestimation of neuronal counts was noticeable in areas with high neuronal density, such as the frontal cortex, whereas a systematic overestimation of neuronal counts occurred in areas with low neuronal density due to the inclusion of segments of dendrites as purported neurons. To avoid these biases, a separate strategy was pursued for neuron segmentation in single-brain ROIs alone. RGB images with single region outlines were generated as explained in https://gitter.im/tractatus/Lobby and imported in the platform *Fiji* [39]. Another *Fiji* macro was then designed to mask and resize the RGB image to match the original high-resolution image. An outline of the brain ROI, based on the Hue-saturation-brightness color model stack, was created in the RGB image and recovered in the original high-resolution image to crop.

The *ilastik* toolkit [42] was subsequently used. The Pixel Classification workflow of *ilastik* enabled discrimination of neurons (foreground) from neuron-devoid brain architecture (background); the resulting binary images (Fig. 1a, 4.3) were fed to a final *Fiji* macro to crop highly-resolved brain ROIs (Fig. 1a, 4.2) to smaller ROIs containing only neurons (Fig. 1a, 4.6) while excluding brain ROIs without LHA-projecting somata from further analysis. This step proved crucial to resort to a highly trained Cell Density Counting suite in *ilastik*, since brain architecture removal reduced the chance of generating false positives and drastically reduced the burden on the random-access memory.

### Ilastik training

For both Pixel Classification and Cell Density Counting workflows, σ values of 1.0, 1.6, 3.5 and 5.0 were selected for the following features: Gaussian Smoothing, Laplacian of Gaussian, Gaussian Gradient Magnitude, Difference of Gaussian, Structure Tensor Eigenvalues, and Hessian of Gaussian Eigenvalues. Twenty randomly selected whole-section images were sufficient for Pixel Classification training; batch processing generated simple-segmentation .tiff images (Fig. 1a, 4.3).

On the other hand, the Cell Density Counting classifier required a total of 250 randomly selected images for training. As displayed in Fig. 1b, after annotating 100 images the training effect had already reached saturation. Additional 150 images were annotated to produce a higher accuracy. Furthermore, we adapted the parameters of the implemented Random Regression Forest algorithm (described in more detail in [43]), i.e., the number and the maximum depth of the trees (Ntrees = 20, MaxDepth = 80), and achieved a final accuracy of 94% by comparing counts to a selection of 100 randomly selected and manually annotated images. Random sampling of probability maps exported upon batch image processing provided a further quality control (Fig. 1a, 5.2).

### Determination of single brain regions and injection site volumes

Volume measurements were performed with yet another custom macro in *Fiji*, measuring the area of a scaled and cropped image in TIFF format (Fig. 1a, 4.2) and multiplying it by the thickness of the section (70 μm). The injection site volume was determined by manually defining their cross-section in every brain slice at a set image contrast and once again multiplying the area by the section thickness. Brains with injection site volume > 1 mm^3^, with a mislocalized position or displaying a virus backflow (visible in dorsal regions with respect to the LHA) > 10% of the whole injection site were excluded from further analysis.

### Determination of misfSOD1 burden

A dedicated macro in *Fiji* was used to measure the area occupied by misfSOD1-positive cells defined upon RenyiEntropy thresholding [44] and subsequent background subtraction (rolling ball radius = 20 px).

### MRI acquisition for hypothalamic volumetry and hypothalamus volume quantification

The T1w MRI data for 72 patients with ALS and 43 controls were acquired using a 3.0 T MRI scanner (Allegra Siemens Medical, Erlangen, Germany) with a T1-weighted magnetisation-prepared gradient echo image (MPRAGE) sequence (192 sagittal slices, no gap, voxel size 1.0 × 1.1 × 1.0 mm^3^, matrix 256 × 192 × 256, TE = 4.7 ms, TR = 2200 ms).

The MPRAGE images were used for manual delineation of the hypothalamus in the coronal plane using a previously reported landmark-based procedure (Tensor Imaging and Fiber Tracking software, TIFT [19]). Briefly, a three-step processing pipeline was used: (1) rigid brain normalisation along the anterior commissure (AC)–posterior commissure (PC) axis; (2) spatial upsampling into a study-specific grid (in-plane resolution 62.5 × 62.5 μm^2^, coronal slice thickness 0.5 mm) to improve the accuracy in identifying landmarks and hypothalamic borders; and (3) delineation of the left and right hemispheric hypothalamus by intensity-threshold-based semi-manual slice-wise identification of hypothalamus in coronal slices.

### MRI acquisition of DTI datasets

DTI scanning was performed in 83 ALS patients (58 ± 14 years; 49 males; ALS Functional Rating Scale–Revised, 40 ± 6; disease duration, 19 ± 16 months) and 64 controls (52 ± 11 years, 33 males) according to a standardized protocol (for details see [45]). The DTI data were acquired on a 3.0 T head scanner (Allegra, Siemens Medical, Erlangen, Germany). The standardized DTI scanning protocol was as follows: 49 gradient directions (b = 1000 s/mm^2^) including one b = 0 gradient direction, 52 slices, 96 × 128 voxels in-plane, slice thickness 2.2 mm, in-plane voxel size 2.2 mm × 2.2 mm, echo time 85 ms, and repetition time 7600 ms. All participating patients and controls provided written informed consent for the study according to institutional guidelines. The study was approved by the Ethical Committee of the University of Ulm (reference #19/12).

### DTI dataset analysis

The TIFT software [46] was used for DTI data processing and statistical analysis, as previously described [45]. In brief, stereotaxic normalization to the Montreal Neurological Institute space was performed iteratively using study-specific templates. To map the white matter microstructure, fractional anisotropy (FA) maps were calculated from the stereotaxically normalized DTI data sets of all subjects. A Gaussian filter of 8-mm full-width at half maximum was applied for smoothing of FA maps for a good balance between sensitivity and specificity [47] and the FA maps were corrected for the covariate age. Tractwise FA statistics was performed by comparing the FA values between the two subject groups in a given tract system (Student’s *t*-test) [48], focusing on the following tract systems: a tract from orbitofrontal regions to the hypothalamus (orbitofrontal-hypothalamic tract), a tract from the hypothalamus to the insula (insular-hypothalamic tract), the cingulate-hypothalamic tract, and the corticospinal tract as a reference. Consequently, a tract-of-interest (TOI) analysis allowed for quantification of microstructural alterations in these tract systems.

### Statistical analysis

Statistical analysis was performed with the GraphPad8 software suite. Comparison of neuronal counts (absolute or normalized value) was performed by two-way ANOVA (genotype x structure design) using Sidak’s *post-hoc* multiple comparisons correction; corrected *P* values are provided. Normalized counts were obtained by dividing the absolute number of neurons in each structure by the total number of neurons counted in the same brain and multiplying the ratio by 50,000 (50 k normalization). To compensate for the atrophied LHA, the individual LHA volume of each mouse was divided by the average LHA volume of wild-type (WT) mice (2.75 mm^3^), and this ratio was multiplied with the absolute number of neurons counted in each area (LHA atrophy compensation). Comparison of the total neuronal counts was performed with the two-tailed unpaired Student’s *t*-test. Evaluation of misfSOD1 burden was performed with the one-way ANOVA with Tukey’s *post-hoc* test.

## Results

### Enhanced semi-automated mouse brain segmentation with neuronal annotation for global quantification of projections with single-cell resolution

**Fig. 2:**
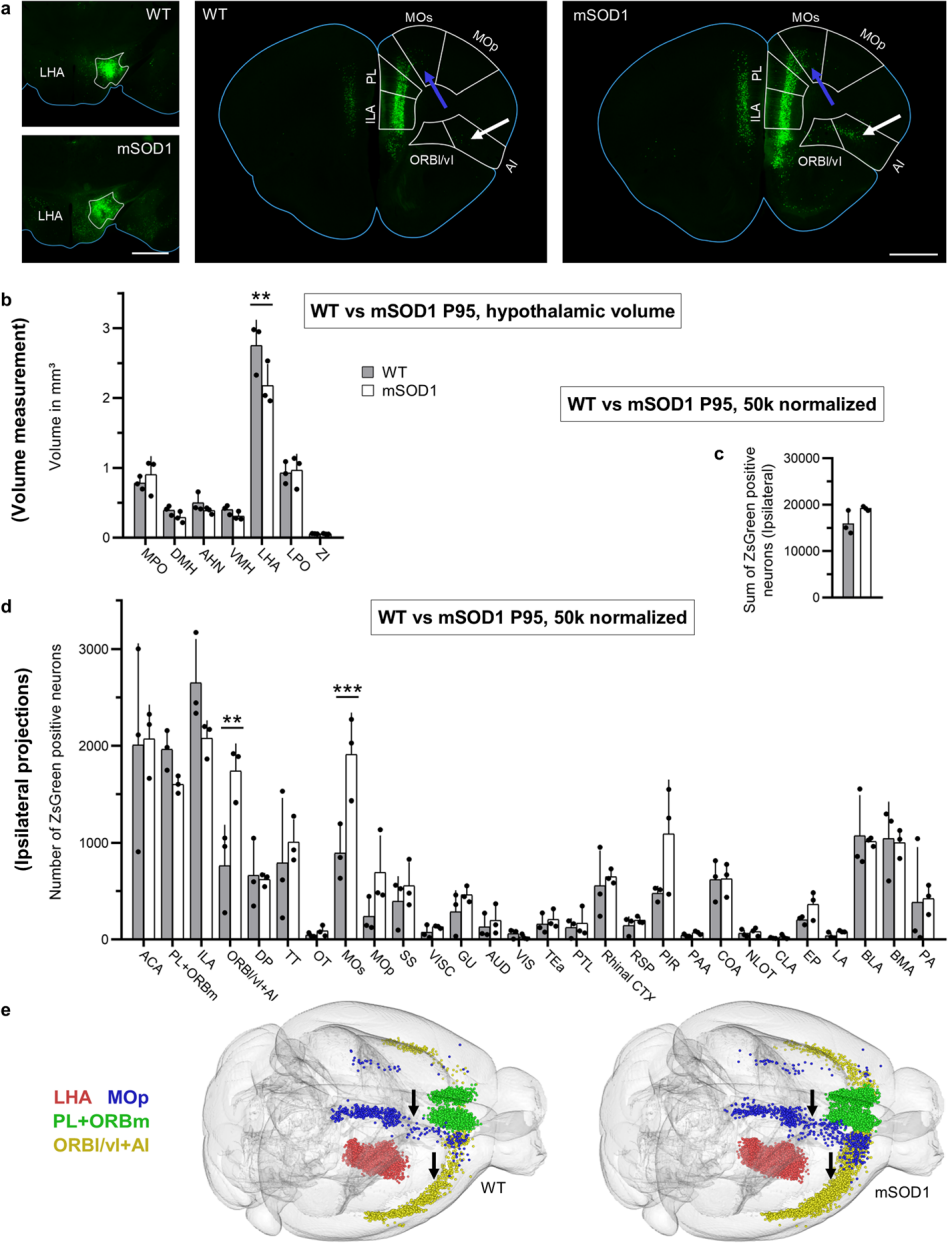
Altered cortico-hypothalamic projection pattern in mSOD1 mice at P95. **a** Left: Representative injection sites in LHA for WT and mSOD1 mice. White outlines represent LHA boundaries. Middle and right: Representative frontal brain sections of WT and mSOD1 mice depicting projections from ORBl/vl + AI (significantly increased, arrow), PL + ORBm, ILA, ACA, MOs and MOp to LHA. **b** Volumetric comparison of seven representative areas of the hypothalamus (HY) in WT and mSOD1 mice. Significant atrophy of the LHA (*P* < 0.000 1) was detected. **c** Sum of neurons projecting from the selected 28 areas. No difference was detected in the number of neurons projecting to the LHA between WT and mSOD1 from the ipsilateral hemisphere (*n* = 3). **d** Number of neurons (normalized for total neuronal counts, 50 k) projecting to the LHA from the 28 brain areas in WT and mSOD1. A significant increase in projections from ORBl/vl + AI (*P* = 0.001 2) and from MOs (*P* = 0.000 6) was detected. **e** Representative *WholeBrain* reconstructions of neurons projecting to the LHA in WT and mSOD1 mice in a brain-wise manner. Projections from MOs (blue) and ORBl/vl + AI (yellow) were increased (arrows indicate the MOs, highlighted in blue, and the ORBl/vl + AI highlighted in yellow). Bars represent mean ± SD. Scale bars, 1 mm. **P < 0.01, ***P < 0.001

To build a quantitative and anatomically accurate map of projections from cortical and subcortical areas to the LHA, we first set out to establish a reliable approach to identify neurons and register their position in the structure classification of the Allen Brain Anatomical Reference Atlas (Fig. 1a). The image batch for software training was obtained by injecting the retrograde rAAV2 (rAAV2-retro) into the LHA of two ZsGreen reporter mice. The mice were sacrificed 15 days later and the fixed brain was serially sectioned at 70 μm thickness; the whole glass slide with mounted sections was scanned using a Leica epifluorescence microscope. Each atlas plate was warped onto the brain section by defining an appropriate number of corresponding anatomical landmarks with *WholeBrain* (see the Methods section), thereby matching the atlas coordinates with the actual brain sections and allowing a proper anatomical annotation (Fig. 1a, 2). Single-structure outlines (cortical layers, subcortical structures, e.g. Fig. 1a, 4.1) were extracted using a dedicated *R* script, then size-corrected and re-overlapped onto the original image in *Fiji*; the corresponding area in the original image was then cropped (Fig. 1a, 4.2), resulting in the unpacking of the original single-section into several-dozen cropped pictures (each image was coded to be identified later), resulting in > 100,000 pictures per brain. To identify and count the number of neurons in each fragment, the *ilastik* software suite was employed. Simple foreground/background segmentations were generated by resorting to the pixel classification workflow, which allowed automatic clearance of most of the brain autofluorescence background in *Fiji* (Fig. 1a, 4.6). This step improved the discrimination of few ZsGreen+ neurons in ROIs containing many more dendrite stretches and artifacts, minimizing the chance of generating artifactual counts and reducing the computational burden placed on *ilastik* cell density classifier. The latter was trained with 250 randomly selected ROI images that were annotated by a human operator; the accuracy of the software reached 86% after training with 100 images and achieved a peak of 94% (Fig. 1b) by improving the performance of the Random Regression Forest algorithm with 150 additional images and tuning its parameters (trees number and depth; see Methods). The whole procedure was then sequentially implemented so that the neuronal counts for each picture and every section were annotated and logged together with their anatomical location within the brain hemisphere as well as with the rostro-caudal serial number of the section itself.

### Disrupted cortico-hypothalamic projections in symptomatic mSOD1 ALS mice

We used our enhanced registration-segmentation-annotation pipeline to establish whether the large-scale architecture of the projections to LHA was altered in the mSOD1 mice at a symptomatic stage (defined by the appearance of motor impairment, according to the progression rate reported previously) [35]. To this aim, 20 mSOD1/ZsGreen+ and 20 WT/ZsGreen+ mice were injected at the age of P95 with rAAV2-retro [36] encoding the Cre protein under the human synapsin promoter in the LHA area (Fig. 2a). All neurons projecting to the site of injection would take up the rAAV2-retro, express the Cre recombinase and appear ZsGreen+. Mice were sacrificed 15 days later. Upon serial sectioning, a series of quality control criteria were applied and brains displaying (i) improper location of the injection site, (ii) injection volume larger than the boundaries of the LHA, (iii) a large number of infected neurons along the thread of the injection capillary, or (iv) a low (< 25,000) overall number of ZsGreen+ neurons were excluded from further analysis. Six brains per genotype were then considered for further quantification. These brains were randomly divided into two cohorts: cohort 1 was subjected to *WholeBrain* parcellation and cohort 2 was left for confirmation (see below). Upon co-registration with the anatomical template in *WholeBrain,* we identified multiple hypothalamic areas (defined according to the ABA); there was a significant difference in the volume of these areas between mSOD1 mice and WT littermates (two-way ANOVA: *F*_1,28_ = 3.663, *P* = 0.065 9), which, as revealed by the *post-hoc* analysis, was due to a significant decrease in the LHA volume (*P* = 0.002 8; Fig. 2b), whereas other hypothalamic areas were comparable in volume between the two groups. This suggests the involvement of the hypothalamic system (which is associated with metabolic dysfunction and reduced BMI in human ALS patients [19]) at this stage of disease in mSOD1 mice. Upon annotation and neuron identification, we counted 50,000 neurons on average that projected to the LHA in WT mice; after the brainstem was excluded (because anatomical annotation of the brainstem nuclei was judged unreliable based on visual inspection of the *WholeBrain*-overlapped images), we identified 84 cerebral areas projecting to the LHA (full list with absolute and 50 k normalized neuronal counts is provided in Table S2). Projections were identified from a large fraction of the forebrain, with the largest contribution provided by limbic areas such as the anterior cingulate area (ACA), prelimbic cortex (PL), infralimbic area (ILA) and agranular insula (AI), as well as by subcortical structures belonging to the limbic system such as the basolateral and basomedial amygdala (BLA and BMA, respectively). Intriguingly, substantial projections were identified from the motor areas (primary and secondary), from primary sensory areas (gustatory, auditory, olfactory/piriform, visual cortex) and from basal ganglia (dorsal putamen), highlighting the breadth of integration in the LHA. The same structures appeared to project to LHA from ipsilateral and contralateral hemispheres, although the contralateral contribution appeared to be substantially lower (about 40,000 neurons from the ipsilateral hemisphere versus 10,000 from the contralateral in WT animals).

The absolute neuronal counts in 28 areas (accounting for ~ 50% of the total projections; excluding areas with counts < 100 neurons) that represent projections were compared between WT and mSOD1 mice in ipsilateral and contralateral sides, respectively, while taking PL and orbitomedial cortex (ORBm), as well as the lateral/ventrolateral orbitofrontal cortex (ORBl/vl) and AI as two wholes due to the uncertainty in establishing their borders and in agreement with functional similarity. While the LHA in symptomatic mSOD1 mice received projections from the same structures as in WT ones, the cumulative absolute counts of ZsGreen+ neurons for the 28 areas were significantly increased in the mSOD cohort (*P* = 0.02; Fig. S1). Two-way ANOVA revealed a significant effect of mSOD1 transgene expression (*F*_1,112_ = 105.70, *P* < 0.000 1) and *post-hoc* analysis (Sidak’s multiple comparisons test) revealed significant expansion of the projections from ACA (*P* < 0.000 1), ILA (*P* = 0.009), ORBl/vl + AI (*P* < 0.000 1), Tenia Tecta (TT; *P* = 0.036 5), secondary motor cortex (MOs, *P* < 0.000 1) and piriform cortex (PIR; *P* = 0.000 5) (Fig. S1b). After compensation for the reduced volume of the LHA (which may affect the density of axonal projections) in the mSOD1 mice, two-way ANOVA still showed a significant effect of mSOD1 expression on the number of projecting neurons/mm^3^ LHA (*F*_1,112_ = 39.35, *P* < 0.000 1) and the *post-hoc* analysis (Sidak) revealed that projections from the ORBl/vl + AI (*P* < 0.000 1), MOs (*P* < 0.000 1) and PIR (*P* = 0.002 31) were significantly expanded in the mSOD1 mice (Fig. S1c). To account for the variable total number of infected neurons in each individual brain, we then normalized the absolute counts of neurons projecting to the LHA to a nominal value of 50,000 neurons, so as to compute the relative contribution of each area to the total input to LHA. Upon 50 k normalization, the sum of neurons in the 28 areas amounted for a comparable number of neurons (Fig. 2c). Two-way ANOVA on 50 k normalized data revealed once again a significant effect of mSOD1 expression (*F*_1,112_ = 7.31, *P* = 0.007 9) and *post-hoc* analysis revealed a significant increase in projections from the ORBl/vl + AI (*P* = 0.001 2) and the MOs (*P* = 0.000 6; Fig. 2d; also visualized in the 2D projections in Fig. S1d and e, as well as in the 3D *Wholebrain* reconstruction in Fig. 2e). The remaining 26 areas displayed non-significant trends toward expanded projections (PIR, *P* = 0.215 2), reduced projection (ILA, *P* = 0.325 6) or no difference in the size of neuronal population projection to the LHA (Fig. 2d). When projections from the contralateral hemisphere were considered, we detected an effect of transgene expression on the absolute counts (two-way ANOVA *F*_1,112_ = 31.49; *P* < 0.000 1) that was traced in *post-hoc* test (Sydak’s) with a significant expansion of projections from ACA (*P* = 0.000 8), ILA and PL + ORBm (both *P* < 0.000 1) (Fig. S2a, b). However, when the absolute counts were compensated for LHA atrophy, the effect of the genotype remained (two-way ANOVA *F*_1,112_ = 7.926 0; *P* = 0.005 8), but once again the *post-hoc* analysis revealed no significant differences in any single area (Fig. S2c). Likewise, when the absolute counts were normalized for 50 k projecting neurons, no significant differences were detected (two-way ANOVA, *F*_1,112_ = 0.310 8, *P* = 0.578 3; Fig. S3a).

Since we had already identified LHA atrophy in mSOD1 and given that mice with neurodegenerative phenotypes may display cortical atrophy [49], we further used the annotation made with *WholeBrain* to measure the volume of each cortical structure and compensate for possible volume loss. Indeed, we detected significant atrophy in mSOD1 mice (two-way ANOVA, *F*_1,112_ = 28.800 0, *P* < 0.000 1), which was traced by *post-hoc* test (Sidak’s) to the primary motor area (MOp) (*P* = 0.008 0) and somatosensory areas (SS) (*P* < 0.000 1), with only a trend for MOs (*P* = 0.335 7; Fig. S3b). Notably, ORBl/vl + AI and MOs did not display significant changes in volume.

These results suggested the occurrence of a substantial remodeling of cortico-hypothalamic projections in P95 mSOD1 mice, in particular projections from ORBl/vl + AI and MOs.

### Validation of cortico-hypothalamic projection abnormalities in independent operator-registered brain cohorts

Since the identification of different cortical and subcortical areas was based on the assumption of overall similarity of brains of mSOD1 and WT mice, it was possible that biases may be introduced into the identification of anatomical areas by *WholeBrain* (e.g., because of atrophy), thereby mis-attributing neurons to the wrong cortical areas. Therefore, we used the brain cohort 2 to validate the findings (*n* = 3 for WT and mSOD1; LHA injection; Fig. S4a). These brain images were subjected to manual registration, i.e., each anatomical structure and cortical area was identified by a genotype-blind operator using structural landmarks (Table S1) and individually cropped out of each brain section image. This approach was substantially slower than the registration *via* *WholeBrain* (> 500 h for a whole dataset). The individually cropped images were then segmented with the Cell Density Counting module in *ilastik* (since the accuracy and reproducibility of this step had already been established and quantified). In the manually annotated dataset, the sum of neurons projecting from the 28 areas to the LHA was not significantly different between mSOD1 and WT (Fig. S4b). First, we considered absolute neuronal counts: the comparison between the two genotypes (ipsilateral hemisphere) revealed a statistically significant difference (two-way ANOVA, *F*_1,112_ = 6.281 0; *P* = 0.013 6), further *post-hoc* comparison (Sydak’s) revealed a significant expansion of projections from the ORBl/vl + AI in mSOD1 mice (*P* = 0.035 2) (Fig. S4c). Upon 50 k normalization we also detected a significant effect of genotype (two-way ANOVA, *F*_1,112_ = 23.780 0; *P* < 0.000 1) with *post-hoc* revealing significant differences in ORBl/vl + AI (*P* < 0.000 1), ILA (*P* = 0.000 1) and PIR (*P* < 0.000 1) (Fig. S4d). However, no significant effect of genotype was found on the absolute counts of neurons projecting from the contralateral hemisphere (Fig. S5a), or in any area of interest (Fig. S5b), but upon 50 k-normalization a statistically significant loss of projections from the PL + ORBm was identified (*P* < 0.000 1), together with a trend toward increase of projections from the ORBl/vl + AI (*P* = 0.072) (Fig. S5c). Thus, the manually registered dataset displayed substantial similarities with the *WholeBrain*-registered dataset (expansion of projections from the ORBl/vl + AI) but also a major discrepancy: increased projection from the ILA to LHA in the manual dataset and no difference in MOs projections, whereas the opposite was detected by *WholeBrain*. Closer inspection of MOs projection to LHA revealed a high degree of subjectivity during manual delineation of the boundaries of MOs and ILA, and it was highly likely that MOs neurons were allocated to adjacent areas and ILA was moved to neighboring areas in the dataset parcellated by the human operator.

### Unaltered projections to the LHA in early presymptomatic mSOD mice

**Fig. 3:**
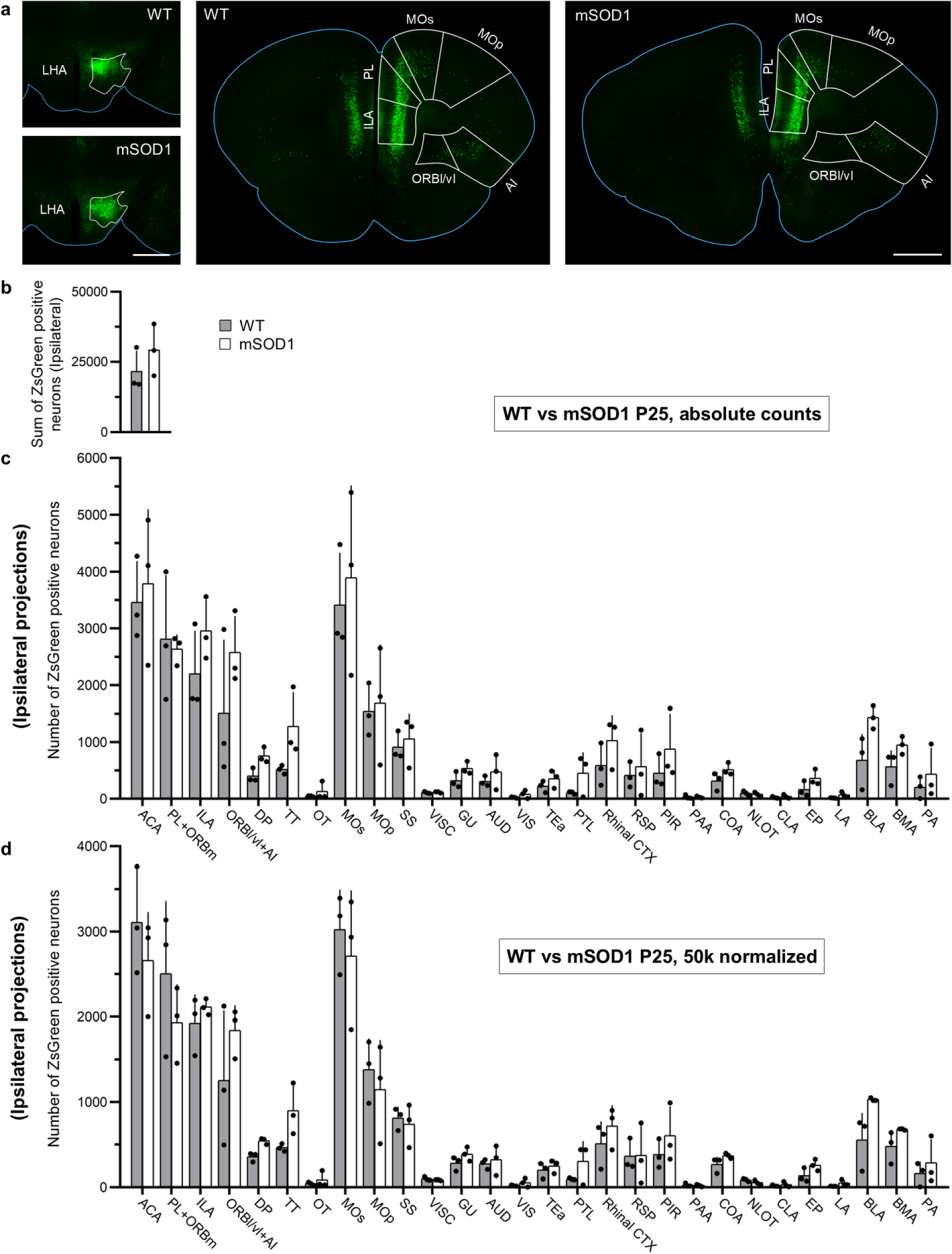
The cortico-hypothalamic projection pattern in mSOD1 mice at P25 at the ipsilateral side was not significantly changed compared to the WT mice. **a** Left: Representative injection sites in the LHA for WT and mSOD1 mice. White outlines represent LHA boundaries. Middle and right: Representative frontal brain sections of WT and mSOD1 mice depicting a similar pattern of neurons. **b** Sum of neurons projecting from the selected 28 areas. No difference was detected in the number of neurons projecting to the LHA in WT and mSOD1 from the ipsilateral hemisphere (*n* = 3). **c** No difference was detected in the absolute number of neurons projecting to the LHA from the 28 brain areas in WT and mSOD1. **d** No difference was detected in the 50 k normalized number of neurons projecting to the LHA from the 28 brain areas in WT and mSOD1. Bars show mean ± SD. Scale bars, 1 mm

To verify if the alterations in projections to LHA occurred with disease progression or had been already present at the earliest stages of disease, we injected rAAV2-retro into the LHA of WT or mSOD1 (*n* = 3 for each) at the age of P25 (Fig. 3a) and sacrificed them at P40. After quality control, we assessed the forebrain projections to LHA. The overall pattern of projections to LHA at P25 was comparable to that observed at P95 and there was no difference in the absolute number of neurons projecting to the LHA between the two genotypes (Fig. 3b). No difference in the volume of any hypothalamic structure, including the LHA, was seen at this stage (two-way ANOVA: *F*_1,49_ = 0.489 1; Sidak’s *post-hoc* analysis, LHA: *P* = 0.979 6). There was an effect of mSOD1 transgene on the absolute projecting neurons from the 28 ipsilateral areas (two-way ANOVA: *F*_1,112_ = 12.550 0; *P* = 0.006) but not on the 50 k normalized dataset (two-way ANOVA: *F*_1,112_ = 1.838 0; *P* = 0.177 9), and in both cases the *post-hoc* comparison did not reveal any significant difference in any of the considered structures between mSOD1 and WT groups (Fig. 3c, d). Likewise, no statistical difference was found on the sum of projections from the contralateral hemisphere in absolute counts (Fig. S6a) and the significant increase in projections from ACA (*P* = 0.007 8; Fig. S6b) was not confirmed upon normalization for 50 k projecting neurons (Fig. S6c). Indeed, no differences were found in the 3D reconstructions either (Fig. S6d). In all, these findings suggest that the alterations of ORBl/vl + AI projection to the LHA arise during disease progression.

### ALS-related pathology in the ORBl/vl + AI of mSOD1 mice

**Fig. 4:**
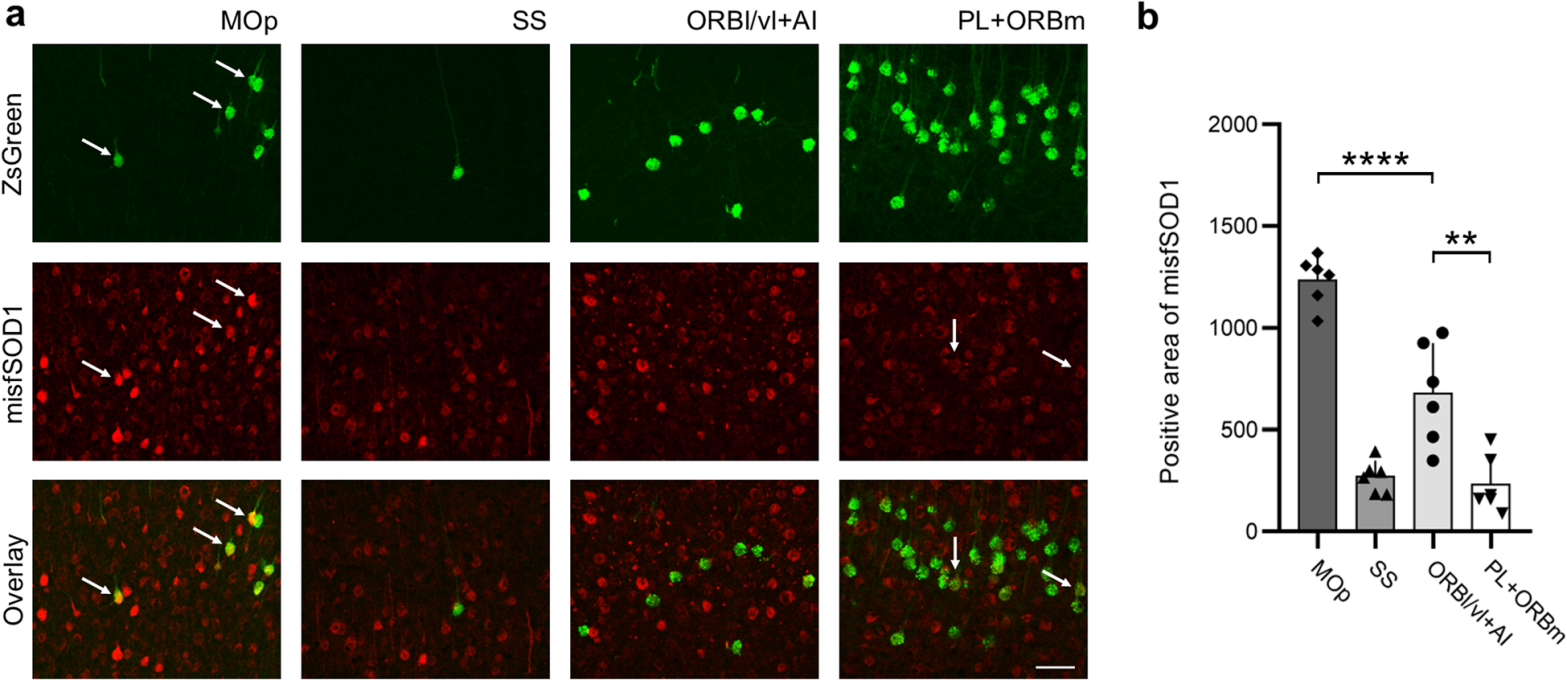
Significant misfolded SOD1 burden in the ORBl/vl + AI in mSOD1 mice. **a** Representative images of misfSOD1+ cells in MOp, SS, ORBl/vl + AI and PL + ORBm; ZsGreen^+^ neurons projecting to the LHA showed a strong misfSOD1 burden (double-positive) in MOp (arrows). Other structures showed no double-positive neurons (SS, ORBl/vl + AI) or weak mSOD burden (PL + ORBm; arrows). **b** Burden of misfolded SOD1 (detected by the B8H10 antibody) in MOp, SS, ORBl/vl + AI and PL + ORBm. The ORBl/vl + AI displayed a significantly higher burden than SS or PL (*P* = 0.001 4 and *P* = 0.000 6 respectively; areas with unchanged connectivity to LHA) but a significantly lower burden than MOp (*P* < 0.000 1). Bars show mean ± SD. Scale bars, 50 μm. **P < 0.01, ****P < 0.000 1

We further investigated the degree of involvement of cortico-hypothalamic projections in ALS by assessing the burden of misfolded SOD1 in cortical areas projecting to LHA, particularly in areas displaying altered connectivity compared to WT animals. Immunostaining of P110 brain sections encompassing the MOp, ORBl/vl + AI, PL + ORBm, and SS showed that the total burden of misfSOD1 was different among the cortical areas (one-way ANOVA, *F*_3,20_ = 51.260 0, *P* < 0.000 1; MOp vs ORBl/vl + AI *P* < 0.000 1, MOp *vs* PL + ORBm *P* < 0.000 1, SS *vs* ORBl/vl + AI *P* = 0.001 4, ORBl/vl + AI *vs* PL + ORBm *P* = 0.000 6). The MOp displayed the highest burden and the ORBl/vl + AI displayed a significantly higher burden than areas showing no increase of projections such as the SS or PL + ORBm (Fig. 4a, b). Interestingly, neurons projecting from MOs to LHA displayed high levels of misfSOD1, while neurons projecting from PL + ORBm and ORBl/vl + AI did not (Fig. 4a).

### Distinct pattern of altered cortical projections to LHA in Fus mice

**Fig. 5:**
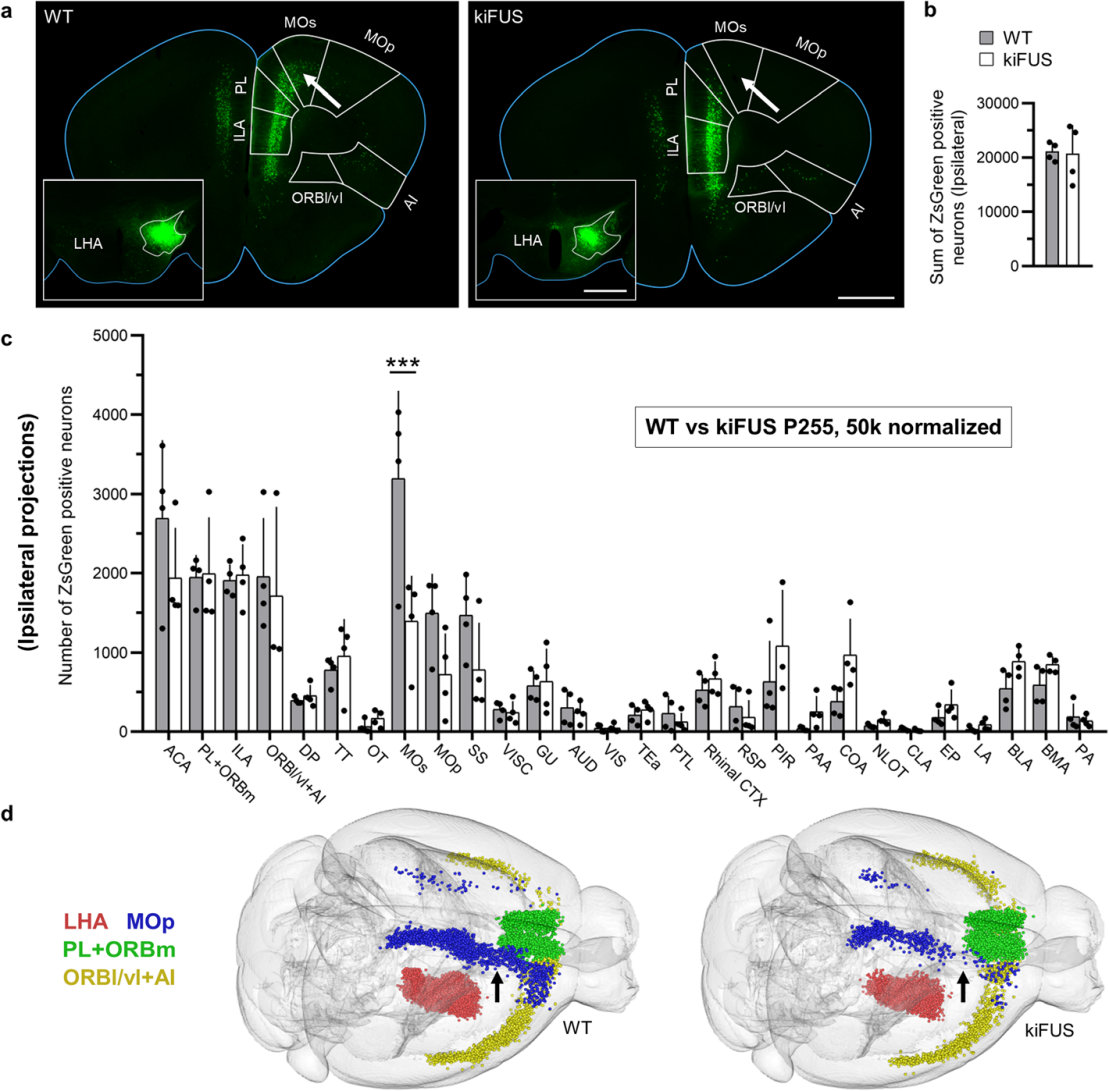
Altered cortico-hypothalamic projection pattern in kiFUS mice at P255. **a** Representative frontal brain sections of WT and kiFUS mice depicting projections from MOs (significantly decreased, arrow), PL + ORBm, ILA, ACA, and MOp to LHA. Inset: representative injection sites in LHA for WT and kiFUS mice. White outlines represent LHA boundaries. **b** Sum of neurons projecting from the selected 28 areas. No difference was detected in the number of neurons projecting to the LHA in WT and kiFUS from the ipsilateral hemisphere (*n* = 4). **c** Number of neurons (normalized for total neuronal counts, 50 k) projecting to the LHA from the 28 brain areas in WT and kiFUS. A significant decrease in projections from MOs (*P* < 0.000 1) and a trend toward decreased projection from ACA (*P* = 0.163 9) and MOp (*P* = 0.136 0) were detected (*n* = 4). **d** Representative *WholeBrain* reconstructions of neurons projecting to the LHA in WT and kiFUS mice. A loss of projections from the anterior part of MOs (blue) could be seen (indicated by black arrow). Bars represent mean ± SD. Scale bars, 1 mm. ***P < 0.001

Next, we aimed at demonstrating that the projection changes observed in the mSOD1 mice are not the consequence of expression of ALS-associated mutations but rather are specific to a model whose phenotype involves body-weight loss and hypermetabolism. We took into consideration the kiFUS ALS model, which does not display an overt body-weight phenotype [50] and shows a more limited loss of spinal motoneurons and slower progression [35]. We proceeded with a similar experimental design as for mSOD1 mice; however, since kiFUS mice do not display spinal motoneuron loss before 9–10 months of age, we considered P255 as an “early symptomatic” stage. Therefore, the kiFUS mice and their WT littermates were injected at P255 into the LHA (Fig. 5a) and sacrificed at P270 (*n* = 4 per group). At this age, we did not detect any change in the volume of the whole hypothalamus or the hypothalamic nuclei (two-way ANOVA, *F*_1,42_ = 1.179 0, *P* = 0.283 7; for LHA, *P* > 0.99; consistent with the absence of body-weight loss reported for this mouse line [50]). There was also no difference in the overall number of neurons projecting to the LHA across the forebrain in absolute counts (Fig. S7a). When the absolute neuronal counts of each of the 28 areas were compared, we detected a significant effect of mutant FUS expression (two-way ANOVA, *F*_1,166_ = 8.583 0; *P* = 0.003 9) and *post-hoc* comparison revealed a significant decrease in projections from ACA (*P* = 0.0385) and MOs (*P* < 0.000 1) in kiFUS mice (Fig. S7b). Upon 50 k normalization, a significant difference in MOs was still detected (*P* < 0.000 1) together with strong trends for ACA (*P* = 0.163 9) and MOp (*P* = 0.136) (Fig. 5b, c; 2D models in Fig. S7c, d). Interestingly, a significant genotype effect was also found in the contralateral hemisphere in both absolute (Fig. S8a, b) and 50 k normalized data (Fig. S8c) (two-way ANOVA, *F*_1,166_ = 6.578 0, *P* = 0.011 2; *F*_1,166_ = 4.488 0, *P* = 0.035 6, respectively), and *post-hoc* analysis revealed loss of projections from ACA (*P* = 0.012 1 and *P* = 0.013 7 in absolute counts and 50 k normalized, respectively) and ILA (*P* = 0.047 4 in the absolute counts only). Of note, projections from the ORBl/vl + AI (either ipsilateral or contralateral) were not affected. The significant loss of MOs neurons projecting to the LHA occurred in the most rostral part of MOs, as demonstrated by the 3D brain reconstruction (Fig. 5d). Thus, distinct ALS models with divergent metabolic phenotype display different patterns of alteration of the LHA inputs.

### DTI-MRI reveals altered orbitofrontal-hypothalamic tract in ALS patients

**Fig. 6:**
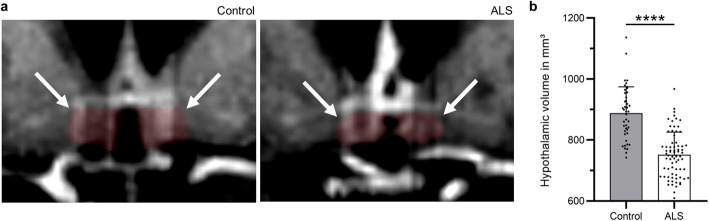
Hypothalamic atrophy in ALS patients. **a** Representative T1w MRI of hypothalami of a healthy control and an ALS patient; hypothalami are shadowed in red (arrows). **b** The hypothalamic volume was significantly decreased in ALS patients (*n* = 72) compared to the controls (*n* = 43). Bars represent mean ± SD. ****P < 0.000 1

**Fig. 7:**
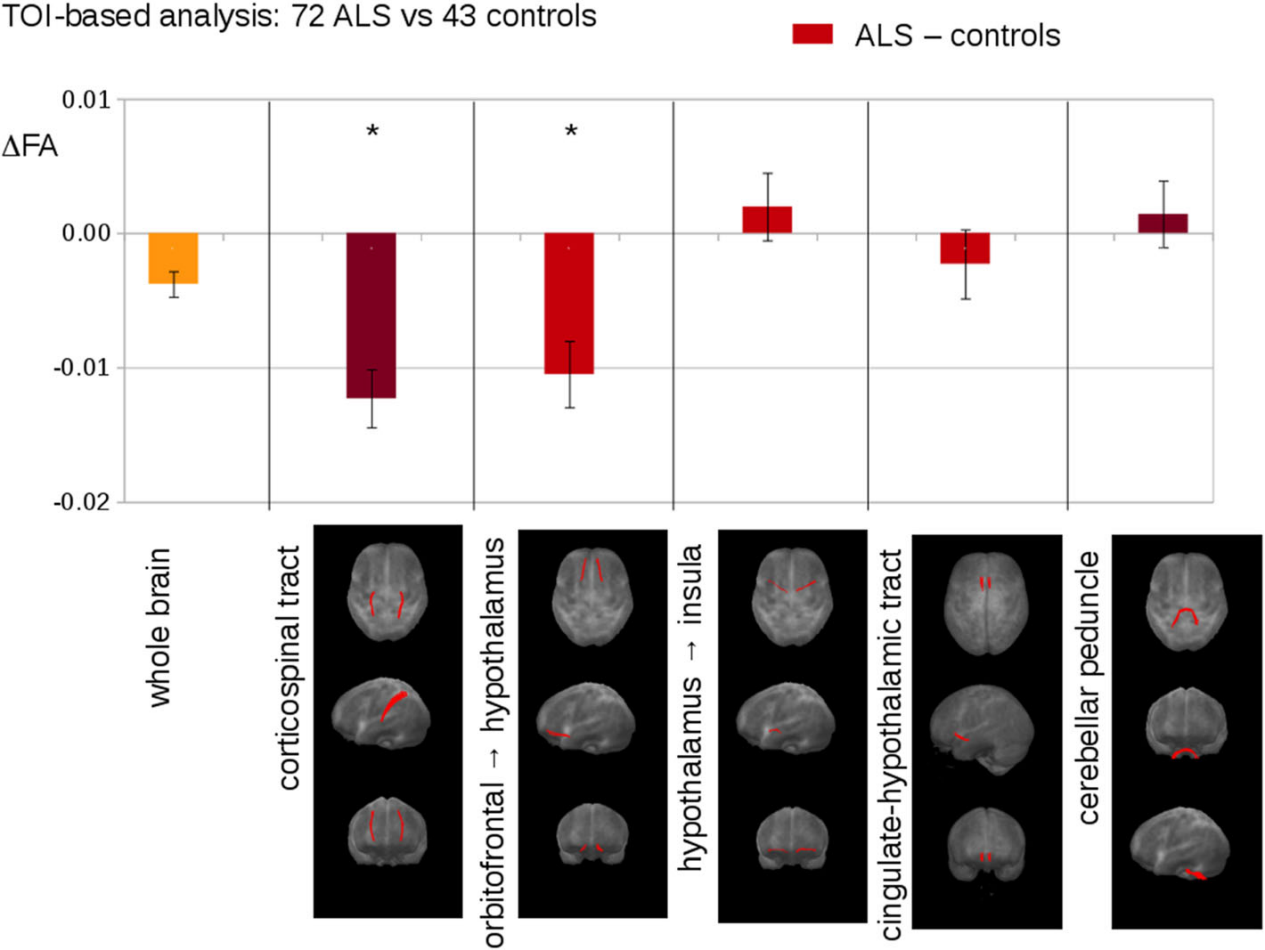
Tract of Interest (TOI)-based analysis of DTI data from 72 ALS patients *vs* 43 healthy controls. Upper panel: differences of the mean fractional anisotropy (FA) values between ALS patients and controls. Lower panel: projectional views (axial, coronal, sagittal) of tract systems used for TOI analysis. Bars represent mean ± SEM. *P < 0.05

To investigate if the selective disturbance in cortico-hypothalamic projections from AI, ORBl/vl and PL observed in the mSOD1 mice also occur in ALS patients, we performed 3 T DTI-MRI on 72 ALS patients and 43 healthy subjects (Table 1 for clinical and demographic characteristics) [45]. Of note, all the ALS patients were sporadic cases and of 33 cases who received genetic screening, only 12 were carriers of mutations in genes causative for ALS (8 C9orf72, 3 SOD1, and 1 FUS). First we verified the hypothalamic involvement in this ALS cohort by demonstrating a significant decrease in hypothalamic volume in the ALS patients (750.8 ± 74.3 mm^3^) compared to the healthy controls (886.9 ± 86.5 mm^3^) (unpaired *t* test: *t* = 8.9, df = 113.0; *P* < 0.000 1; Fig. 6a, b). Then, five white-matter tracts were investigated: the orbitofrontal-hypothalamic tract, the insular-hypothalamic tract, the cingulate-hypothalamic tract (Fig. 7) and, as a reference, the corticospinal tract that is known to be substantially altered in ALS patients [45] and the cerebellar peduncle as control for jitter artifacts. The former three tracts converging on the hypothalamus were selected because they most closely match (when accounting for the different anatomy) the structures investigated in the mouse model and have reproducible and unequivocal identification in DTI datasets. We identified a significant decrease of FA of the orbitofrontal-hypothalamic tract in the ALS patients (∆FA = − 0.011 ± 0.003; *P* < 0.05), with a magnitude comparable to that of the FA loss in the corticospinal tract (∆FA = − 0.013 ± 0.003; mean ± SEM, *P* < 0.001; Fig. 7). For the cingulate-hypothalamic and insular-hypothalamic tracts, small or no FA loss was detected, which did not reach statistical significance. These findings were not due to jitter or motion artifacts as no significant FA alteration was detected in the cerebellar reference (where no ALS affection can be anticipated). Thus, the disturbance of cortical projections to the hypothalamus is not unique to the mSOD1 murine model of ALS but constitutes a previously unrecognized architectural phenotype shared by ALS patients.

**Table 1:** Clinical and demographic characteristics of patients

	*n* (m/f)	Age in years (range)	ALS-FRS-R	Disease duration in months	Genotype
**Controls**	43 (24/19)	56 ± 9 (44–76)	–	–	–
**ALS**	72 (42/30)	58 ± 13 (20–85)	40 ± 6 (23–48)	19 ± 15 (1–60)	8 C9orf72, 3 SOD1, 1 Fus

*ALS-FRS-R* ALS-functional rating scale-revised; *m* male; *f* female

## Discussion

In this study, we provide converging evidence from ALS mouse model and patients of the involvement of large-scale projections to LHA in ALS, in particular the disruption of connections from the orbitofrontal cortex and/or the AI to the LHA. This effect is independent of systematic biases introduced by software or human operators in the registration, does not appear to be an intrinsic developmental defect, and is not detected in an independent ALS model devoid of metabolic phenotypes. The findings in the murine model display striking similarity to the disruption of the orbitofrontal-hypothalamic tract identified in a DTI-MRI dataset of ALS patients.

Hypermetabolism in ALS has been repeatedly reported in a substantial subset of patients [9–13] as well as in some, but not all, ALS murine models [14]. Notably, it may predate the clinical onset of disease by several years [3, 51], and develops over time and correlates with worse prognosis. However, the mechanisms underlying this clinical manifestation are unclear.

The direct involvement of the hypothalamus has been hypothesized to be due to its role as the main regulator of energy homeostasis, feeding and satiety [52] and has been supported by evidence of reduced hypothalamic volume in ALS patients [19, 20] and the detection of ALS-related TDP-43 pathology in the hypothalamus of a subset of ALS patients [53, 54]. Neurochemical abnormalities, such as reduced MCH expression, have been reported in SOD1-mutant murine models [55] and appear to be localized selectively to LHA. However, these signs of intrinsic hypothalamic disturbances are not mutually exclusive, as it has been hypothesized that large-scale hypothalamic circuits may be affected and contribute to the disease. Disruptions of larger circuits regulating the hypothalamic function, which include insular, motor, orbitofrontal and limbic areas, may contribute to the hypothalamic dysregulation. Here we demonstrate that in addition to the appearance of hypothalamic atrophy (detected both in the mSOD1 mice – at the level of the LHA – and in our cohort of ALS patients), the disease pathophysiology also involves a substantial quantitative change in the inputs the hypothalamus receives, particularly from the ORBl/vl and AI.

It should be noted that, in the present study, hypothalamic atrophy was detected at the timepoint of investigation. The relationship between loss of hypothalamus volume and decreased BMI has been previously shown in ALS patients [19] and therefore the hypothalamus volume may act as a proxy of an ongoing disturbance in metabolism regulation.

Notably, the circuit involving projections from the orbitofrontal cortex and AI to the LHA is conserved from mice and rats [56, 57] to marmosets [58] and macaques [59] and to humans, thus validating mouse as a model organism. Nevertheless, the murine orbitofrontal cortex (both ventral and ventrolateral) and AI are considered homologous to the orbitomedial cortex in primates [60], therefore the orbitofrontal-LHA tract in our DTI tracing study is located more medially than the ORBl/vl + AI complex in the mouse brain.

Remarkably, the altered projections from ORBl/vl + AI in mSOD1 mice corresponded to the reduced FA in the orbitofrontal-hypothalamic (but not the insular-hypothalamic) tract in humans. Although the abnormalities occurred in the same brain structures in animal models and patients with ALS, closer examination of the findings reveal that in the murine model, an actual expansion of projections is detected whereas in ALS patients the decreased FA in DTI is usually interpreted as the loss of integrity of axons or myelinated tracts [61]. However, it should be noted that the SOD1^(G93A)^ mice display a fast progression and become symptomatic at a comparatively young age, when the potential of remodeling after degeneration may be larger than that in older human patients.

To the best of our knowledge, there has been no evidence showing that the orbitofrontal cortex and/or AI can directly regulate the metabolic rate. Lesion studies of these structures have not included a rigorous, long-term testing of body-weight dynamics, and inactivation studies have been performed only in short-term settings [62–64]. On the other hand, the orbitofrontal cortex and AI are involved in the processing of food attributes, such as taste, smell and texture [65], and display a differential response to caloric content of food depending on the satiety of the subject (at least in humans [66, 67]). Thus, it is conceivable that the altered connectivity between ORBl/vl + AI and LHA may provide the latter with incorrect inputs regarding the nutritional content of food, contributing to the dysregulation of body metabolic rates. The reduced volume of grey matter and altered microstructural integrity (measured by apparent diffusion coefficient) in the orbitofrontal cortex have been observed in obese patients [68–70], strengthening the link between dysfunction in the orbitofrontal cortex and body weight.

The comparison of mSOD1 and kiFUS models highlight both similarities and discrepancies. On the one hand, the two models differ in the involvement of the hypothalamus itself (no atrophy in the kiFUS) and in the projections to the hypothalamus (consistent with their respective body-weight phenotypes). Compared to the human subjects, the mSOD1 model appears to more closely mimic the patient’s data, while FUS mutations are not only linked to ALS but also to frontotemporal dementia (FTD) [71], as the FUS mutant mice appear to share phenotypes with FTD [72, 73]. Thus, the different patterns of cortical and subcortical vulnerability may determine the appearance of full ALS-like phenotypes. On the other side, the two models show common involvement of projections from the primary and secondary motor cortex to LHA (although with different outcomes), stressing the vulnerability of the motor cortex in both models to the ALS-related pathogenic process. Interestingly, the connectivity between motor cortex and LHA appears to be reciprocal, since LHA projections to motor cortices have also been reported [26]. These connections cannot be easily discounted as artifacts due to the proximity of LHA to the corticospinal tract, since they appear to originate from both ipsilateral and contralateral hemispheres. It is conceivable that LHA receives inputs relaying the average motor activity performed or planned and may adjust energy balance accordingly; disruption of the connectivity between the motor cortex and the hypothalamus may contribute to the dysfunction of the hypothalamic networks. Remarkably, the kiFUS mice display a distinct loss of projections from the motor cortex, which is an interesting phenotype to be further investigated; however, here we have considered the kiFUS model to demonstrate that any projection remodeling is not a simple consequence of ALS-related mutations but rather a selective phenotype of the ALS mouse model displaying body-weight loss and hypermetabolism. The in-depth characterization of the projection architecture of the kiFUS mouse and its evolution over the disease course was not the primary object of this work.

## Conclusion

Our findings suggest that the disruption of large-scale circuits providing input to the LHA contributes to the generation of metabolic phenotype in ALS; in particular, we have identified the orbitofrontal-hypothalamic tract as a site of convergence of mouse projection data and human tracing data, which may open up an independent approach to evaluate non-motor prognostic features in ALS.

## Supplementary Information

**Additional file 1: Supplemental file 1. Table S1.** Anatomical landmarks used to map brain sections along the anterior-posterior axis. **Table S2.** List of brain structures projecting to LHA.

**Additional file 2: Supplemental file 2. Fig. S1.** Altered cortico-hypothalamic projection pattern in mSOD1 mice at P95, absolute ipsilateral counts. **Fig. S2.** Altered cortico-hypothalamic projection pattern in mSOD1 mice at P95, contralateral hemisphere. **Fig. S3.** 50k normalized contralateral projection in P95 mSOD1 animals and MOp/SS atrophy in P95 mSOD mice. **Fig. S4.** Altered cortico-hypothalamic projection pattern in manually registered mSOD1 mice at P95, ipsilateral. **Fig. S5.** Altered projection pattern in manually annotated mSOD1 mice at P95, contralateral. **Fig. S6.** Unaltered cortico-hypothalamic projection pattern in mSOD1 mice at P25, contralateral hemisphere. **Fig. S7.** Altered cortico-hypothalamic projection pattern in kiFUS mice at P255, absolute counts ipsilateral. **Fig. S8.** Altered cortico-hypothalamic projection pattern in kiFUS mice at P255, contralateral hemisphere.

## Data Availability

The datasets used and/or analyzed during the current study are available from the corresponding author on reasonable request.

## Abbreviations

ABA: Allen brain atlas, version 2011
ACA: Anterior cingulate area
AI: Agranular insular area
ALS: Amyotrophic lateral sclerosis
ALS-FRS-R: ALS functional rating scale revised
AP: Anteroposterior
BLA: Basolateral amygdalar nucleus
BMA: Basolateral amygdalar nucleus
DTI: Diffusion-tensor-imaging
FA: Fractional anisotropy
ILA: Infralimbic area
LHA: Lateral hypothalamic area
MO: Motor cortex
MOp: Primary motor area
MOs: Secondary motor area
MRI: Magnetic resonance imaging
ORBl/vl: Orbital area, lateral/ventrolateral part
ORBm: Orbital area, medial part
PIR: Piriform area
PL: Prelimbic area
SS: Somatosensory areas

## References

[1] Hardiman O, Al-Chalabi A, Chio A, Corr EM, Logroscino G, Robberecht W (2017). Amyotrophic lateral sclerosis. Nat Rev Dis Primers.

[2] Dupuis L, Pradat PF, Ludolph AC, Loeffler JP (2011). Energy metabolism in amyotrophic lateral sclerosis. Lancet Neurol.

[3] Peter RS, Rosenbohm A, Dupuis L, Brehme T, Kassubek J, Rothenbacher D, et al. Life course body mass index and risk and prognosis of amyotrophic lateral sclerosis: results from the ALS registry Swabia. Eur J Epidemiol. 2017;32(10):901–8. 10.1007/s10654-017-0318-z 28975435

[4] Gallo V, Wark PA, Jenab M, Pearce N, Brayne C, Vermeulen R, Andersen PM, Hallmans G, Kyrozis A, Vanacore N, Vahdaninia M, Grote V, Kaaks R, Mattiello A, Bueno-de-Mesquita HB, Peeters PH, Travis RC, Petersson J, Hansson O, Arriola L, Jimenez-Martin JM, Tjonneland A, Halkjaer J, Agnoli C, Sacerdote C, Bonet C, Trichopoulou A, Gavrila D, Overvad K, Weiderpass E, Palli D, Quiros JR, Tumino R, Khaw KT, Wareham N, Barricante-Gurrea A, Fedirko V, Ferrari P, Clavel-Chapelon F, Boutron-Ruault MC, Boeing H, Vigl M, Middleton L, Riboli E, Vineis P (2013). Prediagnostic body fat and risk of death from amyotrophic lateral sclerosis: the EPIC cohort. Neurology..

[5] Dupuis L, Corcia P, Fergani A (2008). Dyslipidemia is a protective factor in amyotrophic lateral sclerosis. Neurology..

[6] Dorst J, Kühnlein P, Hendrich C, Kassubek J, Sperfeld AD, Ludolph AC. Patients with elevated triglyceride and cholesterol serum levels have a prolonged survival in amyotrophic lateral sclerosis. J Neurol. 2011;258(4):613–7. 10.1007/s00415-010-5805-z 21128082

[7] Lindauer E, Dupuis L, Müller HP, Neumann H, Ludolph AC, Kassubek J (2013). Adipose tissue distribution predicts survival in amyotrophic lateral sclerosis. PLoS One.

[8] Marin B, Desport JC, Kajeu P, Jesus P, Nicolaud B, Nicol M, et al. Alteration of nutritional status at diagnosis is a prognostic factor for survival of amyotrophic lateral sclerosis patients. J Neurol Neurosurg Psychiatry. 2011;82(6):628–34. 10.1136/jnnp.2010.211474 21097551

[9] Steyn FJ, Ioannides ZA, van Eijk RPA, Heggie S, Thorpe KA, Ceslis A, et al. Hypermetabolism in ALS is associated with greater functional decline and shorter survival. J Neurol Neurosurg Psychiatry. 2018;89(10):1016–23. 10.1136/jnnp-2017-317887 PMC6166607 29706605

[10] Jésus P, Fayemendy P, Nicol M, Lautrette G, Sourisseau H, Preux PM, et al. Hypermetabolism is a deleterious prognostic factor in patients with amyotrophic lateral sclerosis. Eur J Neurol. 2018;25(1):97–104. 10.1111/ene.13468 28940704

[11] Ahmed RM, Irish M, Piguet O, Halliday GM, Ittner LM, Farooqi S, et al. Amyotrophic lateral sclerosis and frontotemporal dementia: distinct and overlapping changes in eating behaviour and metabolism. Lancet Neurol. 2016;15(3):332–42. 10.1016/S1474-4422(15)00380-4 26822748

[12] Desport JC, Torny F, Lacoste M, Preux PM, Couratier P. Hypermetabolism in ALS: correlations with clinical and paraclinical parameters. Neurodegener Dis. 2005;2(3–4):202–7. 10.1159/000089626 16909026

[13] Bouteloup C, Desport JC, Clavelou P, Guy N, Derumeaux-Burel H, Ferrier A, et al. Hypermetabolism in ALS patients: an early and persistent phenomenon. J Neurol. 2009;256(8):1236–42. 10.1007/s00415-009-5100-z 19306035

[14] Dupuis L, Oudart H, René F. Gonzalez de Aguilar JL, Loeffler JP. Evidence for defective energy homeostasis in amyotrophic lateral sclerosis: benefit of a high-energy diet in a transgenic mouse model. Proc Natl Acad Sci U S A. 2004;101(30):11159–64. 10.1073/pnas.0402026101 PMC503756 15263088

[15] Lim MA, Bence KK, Sandesara I, Andreux P, Auwerx J, Ishibashi J, Seale P, Kalb RG. Genetically altering organismal metabolism by leptin-deficiency benefits a mouse model of amyotrophic lateral sclerosis. Hum Mol Genet. 2014;23(18):4995–5008. 10.1093/hmg/ddu214 PMC4140473 24833719

[16] Scaricamazza S, Salvatori I, Giacovazzo G, Loeffler JP, Renè F, Rosina M (2020). Skeletal-muscle metabolic reprogramming in ALS-SOD1G93A mice predates disease onset and is a promising therapeutic target. iScience.

[17] Ludolph AC, Dorst J, Dreyhaupt J, Weishaupt JH, Kassubek J, Weiland U, et al. Effect of high-caloric nutrition on survival in amyotrophic lateral sclerosis. Ann Neurol. 2020;87(2):206–16. 10.1002/ana.25661 31849093

[18] Dorst J, Schuster J, Dreyhaupt J, Witzel S, Weishaupt JH, Kassubek J, et al. Effect of high-caloric nutrition on serum neurofilament light chain levels in amyotrophic lateral sclerosis. J Neurol Neurosurg Psychiatry. 2020;91(9):1007–9. 10.1136/jnnp-2020-323372 32788256

[19] Gorges M, Vercruysse P, Müller HP, Huppertz HJ, Rosenbohm A, Nagel G, et al. Hypothalamic atrophy is related to body mass index and age at onset in amyotrophic lateral sclerosis. J Neurol Neurosurg Psychiatry. 2017;88(12):1033–1041. 10.1136/jnnp-2017-315795 28596251

[20] Gabery S, Ahmed RM, Caga J, Kiernan MC, Halliday GM, Petersén Å. Loss of the metabolism and sleep regulating neuronal populations expressing orexin and oxytocin in the hypothalamus in amyotrophic lateral sclerosis. Neuropathol Appl Neurobiol. 2021. 10.1111/nan.12709. 10.1111/nan.12709 33755993

[21] González JA, Iordanidou P, Strom M, Adamantidis A, Burdakov D. Awake dynamics and brain-wide direct inputs of hypothalamic MCH and orexin networks. Nat Commun. 2016;7(1):11395. 10.1038/ncomms11395 PMC4844703 27102565

[22] Barbier M, Chometton S, Pautrat A, Miguet-Alfonsi C, Datiche F, Gascuel J, et al. A basal ganglia-like cortical-amygdalar-hypothalamic network mediates feeding behavior. Proc Natl Acad Sci U S A. 2020;117(27):15967–76. 10.1073/pnas.2004914117 PMC7354999 32571909

[23] Barbier M, González JA, Houdayer C, Burdakov D, Risold PY, Croizier S. Projections from the dorsomedial division of the bed nucleus of the stria terminalis to hypothalamic nuclei in the mouse. J Comp Neurol. 2020. 10.1002/cne.24988. 10.1002/cne.24988 PMC7891577 32678476

[24] Murata K, Kinoshita T, Fukazawa Y, Kobayashi K, Kobayashi K, Miyamichi K, et al. GABAergic neurons in the olfactory cortex projecting to the lateral hypothalamus in mice. Sci Rep. 2019;9(1):7132. 10.1038/s41598-019-43580-1 PMC6509143 31073137

[25] Berthoud HR. Multiple neural systems controlling food intake and body weight. Neurosci Biobehav Rev. 2002;26(4):393–428. 10.1016/s0149-7634(02)00014-3 12204189

[26] Commisso B, Ding L, Varadi K, Gorges M, Bayer D, Boeckers TM, et al. Stage-dependent remodeling of projections to motor cortex in ALS mouse model revealed by a new variant retrograde-AAV9. Elife. 2018;7:e36892. 10.7554/eLife.36892 PMC6125125 30136928

[27] Agosta F, Canu E, Valsasina P, Riva N, Prelle A, Comi G, et al. Divergent brain network connectivity in amyotrophic lateral sclerosis. Neurobiol Aging. 2013;34(2):419–27. 10.1016/j.neurobiolaging.2012.04.015 22608240

[28] Schulthess I, Gorges M, Müller HP, Lulé D, Del Tredici K, Ludolph AC, et al. Functional connectivity changes resemble patterns of pTDP-43 pathology in amyotrophic lateral sclerosis. Sci Rep. 2016;6(1):38391. 10.1038/srep38391 PMC5144012 27929102

[29] Heimrath J, Gorges M, Kassubek J, Müller HP, Birbaumer N, Ludolph AC, et al. Additional resources and the default mode network: evidence of increased connectivity and decreased white matter integrity in amyotrophic lateral sclerosis. Amyotroph Lateral Scler Frontotemporal Degener. 2014;15(7–8):537–45. 10.3109/21678421.2014.911914 24862983

[30] Dukic S, McMackin R, Buxo T, Fasano A, Chipika R, Pinto-Grau M, et al. Patterned functional network disruption in amyotrophic lateral sclerosis. Hum Brain Mapp. 2019;40(16):4827–42. 10.1002/hbm.24740 PMC6852475 31348605

[31] Braak H, Brettschneider J, Ludolph AC, Lee VM, Trojanowski JQ, Del Tredici K. Amyotrophic lateral sclerosis--a model of corticofugal axonal spread. Nat Rev Neurol. 2013;9(12):708–14. 10.1038/nrneurol.2013.221 PMC3943211 24217521

[32] Brettschneider J, Del Tredici K, Toledo JB, Robinson JL, Irwin DJ, Grossman M, et al. Stages of pTDP-43 pathology in amyotrophic lateral sclerosis. Ann Neurol. 2013;74(1):20–38. 10.1002/ana.23937 PMC3785076 23686809

[33] Gurney ME, Pu H, Chiu AY, Dal Canto MC, Polchow CY, Alexander DD, et al. Motor neuron degeneration in mice that express a human Cu,Zn superoxide dismutase mutation. Science. 1994;264(5166):1772–5. 10.1126/science.8209258 8209258

[34] Scekic-Zahirovic J, Sendscheid O, El Oussini H, Jambeau M, Sun Y, Mersmann S, et al. Toxic gain of function from mutant FUS protein is crucial to trigger cell autonomous motor neuron loss. EMBO J. 2016;35(10):1077–97. 10.15252/embj.201592559 PMC4868956 26951610

[35] Ouali Alami N, Schurr C, Olde Heuvel F, Tang L, Li Q, Tasdogan A, et al. NF-κB activation in astrocytes drives a stage-specific beneficial neuroimmunological response in ALS. EMBO J. 2018;37(16):e98697. 10.15252/embj.201798697 PMC6092622 29875132

[36] Tervo DG, Hwang BY, Viswanathan S, Gaj T, Lavzin M, Ritola KD, et al. A designer AAV variant permits efficient retrograde access to projection neurons. Neuron. 2016;92(2):372–82. 10.1016/j.neuron.2016.09.021 PMC5872824 27720486

[37] Madisen L, Garner AR, Shimaoka D, Chuong AS, Klapoetke NC, Li L, et al. Transgenic mice for intersectional targeting of neural sensors and effectors with high specificity and performance. Neuron. 2015;85(5):942–58. 10.1016/j.neuron.2015.02.022 PMC4365051 25741722

[38] Paxinos G, Franklin KBJ. The mouse brain in stereotaxic coordinates. 2nd ed. San Diego: Academic Press; 2001. ISBN 0125476361, 9780125476362.

[39] Schindelin J, Arganda-Carreras I, Frise E, Kaynig V, Longair M, Pietzsch T, et al. Fiji: an open-source platform for biological-image analysis. Nat Methods. 2012;9(7):676–82. 10.1038/nmeth.2019 PMC3855844 22743772

[40] Fürth D, Vaissière T, Tzortzi O, Xuan Y, Märtin A, Lazaridis I, et al. An interactive framework for whole-brain maps at cellular resolution. Nat Neurosci. 2018;21(1):139–49. 10.1038/s41593-017-0027-7 PMC5994773 29203898

[41] Lein ES, Hawrylycz MJ, Ao N, Ayres M, Bensinger A, Bernard A, et al. Genome-wide atlas of gene expression in the adult mouse brain. Nature. 2007;445(7124):168–76. 10.1038/nature05453 17151600

[42] Berg S, Kutra D, Kroeger T, Straehle CN, Kausler BX, Haubold C, et al. Ilastik: interactive machine learning for (bio) image analysis. Nat Methods. 2019;16(12):1226–32. 10.1038/s41592-019-0582-9 31570887

[43] Fiaschi L, Koethe U, Nair R, Hamprecht FA. Learning to count with regression forest and structured labels. Proceedings of the 21st International Conference on Pattern Recognition (ICPR2012). Tsukuba. 2012;2685–2688.

[44] Sahoo P, Wilkins C, Yeager J (1997). Threshold selection using Renyi's entropy. Pattern Recogn.

[45] Kassubek J, Müller HP, Del Tredici K, Lulé D, Gorges M, Braak H (2018). Imaging the pathoanatomy of amyotrophic lateral sclerosis in vivo: targeting a propagation-based biological marker. J Neurol Neurosurg Psychiatry.

[46] Müller HP, Unrath A, Ludolph AC, Kassubek J (2007). Preservation of diffusion tensor properties during spatial normalization by use of tensor imaging and fibre tracking on a normal brain database. Phys Med Biol.

[47] Müller HP, Del Tredici K, Lulé D, Müller K, Weishaupt JH, Ludolph AC, et al. In vivo histopathological staging in C9orf72-associated ALS: a tract of interest DTI study. Neuroimage Clin. 2020;27:102298. 10.1016/j.nicl.2020.102298 PMC7270604 32505118

[48] Müller HP, Unrath A, Sperfeld AD, Ludolph AC, Riecker A, Kassubek J (2007). Diffusion tensor imaging and tractwise fractional anisotropy statistics: quantitative analysis in white matter pathology. Biomed Eng Online.

[49] Petrik MS, Wilson JM, Grant SC, Blackband SJ, Tabata RC, Shan X (2007). Magnetic resonance microscopy and immunohistochemistry of the CNS of the mutant SOD murine model of ALS reveals widespread neural deficits. NeuroMolecular Med.

[50] Scekic-Zahirovic J, Oussini HE, Mersmann S, Drenner K, Wagner M, Sun Y, et al. Motor neuron intrinsic and extrinsic mechanisms contribute to the pathogenesis of FUS-associated amyotrophic lateral sclerosis. Acta Neuropathol. 2017;133(6):887–906. 10.1007/s00401-017-1687-9 PMC5427169 28243725

[51] Mariosa D, Beard JD, Umbach DM, Bellocco R, Keller J, Peters TL, et al. Body mass index and amyotrophic lateral sclerosis: a study of US military veterans. Am J Epidemiol. 2017;185(5):362–71. 10.1093/aje/kww140 PMC5860019 28158443

[52] Vercruysse P, Vieau D, Blum D, Petersén Å, Dupuis L (2018). Hypothalamic alterations in neurodegenerative diseases and their relation to abnormal energy metabolism. Front Mol Neurosci.

[53] Cykowski MD, Takei H, Schulz PE, Appel SH, Powell SZ. TDP-43 pathology in the basal forebrain and hypothalamus of patients with amyotrophic lateral sclerosis. Acta Neuropathol Commun. 2014;2:171. 10.1186/s40478-014-0171-1 PMC4297460 25539830

[54] Dedeene L, Van Schoor E, Vandenberghe R, Van Damme P, Poesen K, Thal DR (2019). Circadian sleep/wake-associated cells show dipeptide repeat protein aggregates in C9orf72-related ALS and FTLD cases. Acta Neuropathol Commun.

[55] Vercruysse P, Sinniger J, El Oussini H, Scekic-Zahirovic J, Dieterlé S, Dengler R (2016). Alterations in the hypothalamic melanocortin pathway in amyotrophic lateral sclerosis. Brain..

[56] Floyd NS, Price JL, Ferry AT, Keay KA, Bandler R (2001). Orbitomedial prefrontal cortical projections to hypothalamus in the rat. J Comp Neurol.

[57] Hoover WB, Vertes RP (2011). Projections of the medial orbital and ventral orbital cortex in the rat. J Comp Neurol.

[58] Roberts AC, Tomic DL, Parkinson CH, Roeling TA, Cutter DJ, Robbins TW, et al. Forebrain connectivity of the prefrontal cortex in the marmoset monkey (Callithrix jacchus): an anterograde and retrograde tract-tracing study. J Comp Neurol. 2007;502(1):86–112. 10.1002/cne.21300 17335041

[59] Ongür D, An X, Price JL (1998). Prefrontal cortical projections to the hypothalamus in macaque monkeys. J Comp Neurol.

[60] Price JL (2007). Definition of the orbital cortex in relation to specific connections with limbic and visceral structures and other cortical regions. Ann N Y Acad Sci.

[61] Alexander AL, Lee JE, Lazar M, Field AS (2007). Diffusion tensor imaging of the brain. Neurotherapeutics..

[62] Kobayashi M (2011). Macroscopic connection of rat insular cortex: anatomical bases underlying its physiological functions. Int Rev Neurobiol.

[63] Izquierdo A (2017). Functional heterogeneity within rat orbitofrontal cortex in reward learning and decision making. J Neurosci.

[64] Rolls ET (2004). The functions of the orbitofrontal cortex. Brain Cogn.

[65] Seabrook LT, Borgland SL (2020). The orbitofrontal cortex, food intake and obesity. J Psychiatry Neurosci.

[66] Schur EA, Kleinhans NM, Goldberg J, Buchwald D, Schwartz MW, Maravilla K (2009). Activation in brain energy regulation and reward centers by food cues varies with choice of visual stimulus. Int J Obes.

[67] Suzuki S, Cross L, O'Doherty JP (2017). Elucidating the underlying components of food valuation in the human orbitofrontal cortex. Nat Neurosci.

[68] Raji CA, Ho AJ, Parikshak NN, Becker JT, Lopez OL, Kuller LH, et al. Brain structure and obesity. Hum Brain Mapp. 2010;31(3):353–64. 10.1002/hbm.20870 PMC2826530 19662657

[69] Walther K, Birdsill AC, Glisky EL, Ryan L (2010). Structural brain differences and cognitive functioning related to body mass index in older females. Hum Brain Mapp.

[70] Alkan A, Sahin I, Keskin L, Cikim AS, Karakas HM, Sigirci A, et al. Diffusion-weighted imaging features of brain in obesity. Magn Reson Imaging. 2008;26(4):446–50. 10.1016/j.mri.2007.10.004 18063337

[71] Abramzon YA, Fratta P, Traynor BJ, Chia R (2020). The overlapping genetics of amyotrophic lateral sclerosis and frontotemporal dementia. Front Neurosci.

[72] Ho WY, Agrawal I, Tyan SH, Sanford E, Chang WT, Lim K, et al. Dysfunction in nonsense-mediated decay, protein homeostasis, mitochondrial function, and brain connectivity in ALS-FUS mice with cognitive deficits. Acta Neuropathol Commun. 2021;9(1):9. 10.1186/s40478-020-01111-4 PMC7789430 33407930

[73] de Munter J, Babaevskaya D, Wolters EC, Pavlov D, Lysikova E. Kalueff AV, et al. molecular and behavioural abnormalities in the FUS-tg mice mimic frontotemporal lobar degeneration: effects of old and new anti-inflammatory therapies. J Cell Mol Med. 2020;24(17):10251–7. 10.1111/jcmm.15628 PMC7520339 32667139

