# Supplementary material for: Disruption of orbitofrontal-hypothalamic projections in a murine ALS model and in human patients
Source: Transl Neurodegener. 2021 May 31;10:17. doi: 10.1186/s40035-021-00241-6 (PMC8168014; doi:10.1186/s40035-021-00241-6)
Supplement: Supplementary file 1 — Additional file 1: Supplemental file 1. Table S1. Anatomical landmarks used to map brain sections along the anterior-posterior axis. Table S2. List of brain structures projecting to LHA. [file 40035_2021_241_MOESM1_ESM.pdf]

Table S1: Anatomical landmarks used to map brain sections along the anterior-posterior axis.

Coordinate from bregma	Anatomical landmark	Section in Allen´s brain atlas
2.6	first section without AOB	27
2.2	section before beginning of DP	32
2.1	first section with DP	33
1.6	shape of fa	38
1.5	shape of fa	39
1.4	shape of fa	40
1.2	shape of fa	42
1.1	shape of fa	43
1	shape of ccg	44
0.2	shape of aco	52
0.1	shape of aco	53
0	shape of aco and act	54
-0.3	first section with SCH<br>last section without NLOT	57
-1	shape of opt<br>last section with NLOT	64
-1.4	first section with ME<br>shape of otp and int	69
-2.2	first section without ME<br>first section with ventral DG	76
-2.5	Expansion of CA3so	79
-3.1	last section with MM	85

The following structures provided distinctive optical characteristics for mapping: AOB = Accessory olfactory bulb; DP = Dorsal peduncular area; fa = corpus callosum, anterior forceps; ccg = genu of corpus callosum; aco = anterior commissure, olfactory limb; act = anterior commissure, temporal limb; SCH = Suprachiasmatic nucleus; NLOT = Nucleus of the lateral olfactory tract; ME = Median eminence; opt = optic tract; int = internal capsule; DG = Dentate gyrus; CA3so = Field CA3, stratum oriens; MM = Medial mammillary nucleus.

Table S2: List of brain structures projecting to the LHA.

	**Ipsilateral raw data**	**Contralateral raw data**	**Ipsilateral normalized**	**Contralateral normalized**
**A13**	85.6, ±45.9	10.4, ±6.6	58.5, ±28.2	7.1, ±3.8
**AAA**	297.6, ±230.0	18.8, ±7.2	225.3, ±128.1	16.3, ±7.4
**ACA**	2686.2, ±1373.5	897.0, ±468.9	1826.0, ±538.9	599.4, ±232.6
**ACB**	565.4, ±375.1	9.4, ±10.1	411.8, ±173.1	7.9, ±6.9
**ADP**	64.4, ±27.3	16.6, ±8.6	48.3, ±10.8	12.5, ±4.1
**AHN**	1174.0, ±1241.1	111.4, ±91.1	909.7, ±1146.0	84.7, ±77.7
**AI**	1437.8, ±1365.3	197.0, ±177.6	935.9, ±622.2	117.2, ±82.2
**AON**	92.2, ±90.1	6.2, ±4.4	64.6, ±41.4	4.1, ±1.6
**APN**	54.2, ±43.3	5.4, ±1.7	35.4, ±19.1	5.7, ±4.8
**AUD**	228.6, ±246.6	62.2, ±48.6	138.4, ±129.3	38.0, ±27.4
**AVP**	207.8, ±99.8	71.6, ±36.0	143.2, ±41.5	49.3, ±22.1
**AVPV**	242.0, ±43.6	71.2, ±31.5	216.0, ±99.8	70.5, ±57.7
**BLA**	1263.0, ±670.4	79.6, ±56.3	920.2, ±278.5	52.6, ±28.0
**BMA**	1405.0, ±744.5	110.4, ±67.5	1009.6, ±381.6	77.4, ±37.9
**BST**	2108.8, ±774.0	275.6, ±165.1	1613.9, ±236.4	188.0, ±82.2
**CEA**	440.4, ±315.9	14.4, ±6.8	292.6, ±129.9	12.6, ±7.0
**CLA**	20.0, ±11.5	4.4, ±2.2	14.8, ±4.9	3.8, ±2.1
**CM**	132.8, ±65.5	49.2, ±18.7	97.1, ±40.2	37.5, ±7.1
**COA**	730.4, ±377.3	39.2, ±31.1	539.2, ±179.2	25.4, ±17
**CP**	814.4, ±499.4	34.8, ±21.2	581.7, ±185.8	23.0, ±7.0
**DMH**	1510.0, ±729.1	233.8, ±115.7	1039.7, ±290.7	160.3, ±50
**DP**	618.4, ±249.1	192.2, ±128.9	535.8, ±289.4	131.5, ±54.3

**ECT**	255.8, ±139.4	79.0, ±50.1	172.5, ±48.9	50.5, ±26.3
**ENTl**	144.8, ±96.2	28.6, ±18.1	98.2, ±46.0	18.1, ±10.3
**EP**	252.0, ±241.5	9.4, ±8.1	175.9, ±109.7	5.8, ±4.8
**FS**	59.8, ±45.7	1.0, ±0.9	48.2, ±31.9	0.7, ±0.7
**GP**	133.8, ±83.9	9.8, ±10.7	90.2, ±27.0	6.0, ±5.3
**GU**	503.2, ±399.3	77.0, ±63.8	343.9, ±186.6	49.7, ±28.3
**IA**	127.4, ±90.9	8.0, ±5.8	92.8, ±70.4	6.5, ±5.0
**ILA**	2627.2, ±896.1	1192.2, ±548.6	2089.0, ±476.3	834.9, ±188.3
**IMD**	145.8, ±74.4	55.8, ±29.8	103.6, ±34.6	37.5, ±11.9
**LH**	188.4, ±99.4	32.6, ±17.2	127.9, ±41.1	21.8, ±9.0
**LHA**	Injection	845.8, ±509.0	Injection	573.8, ±167.2
**LPO**	1251.6, ±447.7	173.2, ±113.4	1000.5, ±271.0	120.5, ±51.3
**LS**	1453.4, ±583.2	179.6, ±78.9	1169.3, ±436.5	127.3, ±23.3
**MA**	139.6, ±177.3	5.8, ±4.1	97.8, ±90.1	6.0, ±6.1
**MEA**	712.8, ±691.0	72.4, ±103.5	499.2, ±437.3	46.6, ±66.8
**MEPO**	252.8, ±96.0	204.6, ±71.7	207.2, ±71.9	169.9, ±57.2
**MM**	126.0, ±105.5	55.2, ±49.2	86.8, ±54.7	36.4, ±29.1
**MOp**	1006.4, ±1321.7	71.6, ±74.0	576.5, ±705.8	40.4, ±38.2
**MOs**	2292.6, ±2310.5	273.6, ±224.4	1391.5, ±1146.2	168.8, ±103.4
**MPN**	701.2, ±246.6	177.2, ±104.2	551.6, ±124.7	121.4, ±56.4
**MPO**	1752.4, ±543.1	436.4, ±213.4	1384.5, ±259.7	306.7, ±83.4
**MS**	323.0, ±157.8	58.4, ±29.3	240.5, ±56.7	47.0, ±16.7
**NDB**	288.2, ±281.3	34.4, ±24.5	202.7, ±126.0	24.7, ±20.6
**NLOT**	74.0, ±40.3	8.0, ±5.4	55.9, ±20.7	5.5, ±3.1
**OLF**	125.4, ±76.6	19.0, ±11.4	97.4, ±39.3	13.5, ±4.9

**ORBl**	306.6, ±425.5	41.8, ±68.5	168.7, ±213.7	22.7, ±34.6
**ORBm**	515.4, ±261.3	142.6, ±120.6	421.2, ±183.3	104.2, ±53.7
**ORBvl**	184.8, ±193.9	31.4, ±42.7	110.2, ±90.7	18.6, ±20.7
**OT**	58.4, ±74.6	1.8, ±1.7	37.9, ±35.2	1.5, ±1.0
**OV**	28.2, ±9.0	17.2, ±4.2	24.9, ±12.4	16.2, ±9.8
**PA**	398.4, ±541.0	156.2, ±271.9	252.9, ±347.2	99.8, ±175.5
**PAA**	31.2, ±11.4	2.6, ±0.8	25.3, ±7.4	2.5, ±1.5
**PD**	17.8, ±7.0	3.6, ±3.8	14.7, ±5.9	2.6, ±2.3
**PERI**	235.0, ±141.4	60.8, ±34.0	156.4, ±61.6	39.9, ±15.5
**PH**	1304.0, ±428.6	662.0, ±209.6	1043.6, ±248.9	528.6, ±123.8
**PIR**	667.0, ±677.1	27.0, ±12.9	444.4, ±302.3	20.2, ±6.7
**PL**	1576.0, ±554.1	653.6, ±330.0	1239.8, ±254.5	466.9, ±93.6
**PS**	33.2, ±20.0	2.8, ±2.5	22.2, ±6.3	1.8, ±1.5
**PST**	37.0, ±26.9	4.0, ±4.6	41.3, ±47.5	2.9, ±2.5
**PSTN**	160.2, ±59.7	16.6, ±12.9	168.5, ±158.2	11.4, ±5.6
**PTL**	199.4, ±198.2	17.0, ±11.1	121.9, ±104.4	11.2, ±5.0
**PV**	239.8, ±164.1	46.2, ±27.2	171.9, ±111.7	34.1, ±14.2
**PVH**	449.2, ±210.4	72.4, ±46.7	339.2, ±164.4	50.9, ±33.3
**PVT**	960.4, ±475.5	332.4, ±231.1	686.5, ±334.9	242.4, ±193.1
**RE**	461.0, ±379.7	176.4, ±122.5	356.0, ±347.2	134.9, ±109.3
**RSP**	331.8, ±297.9	47.6, ±32.1	198.7, ±152.4	30.7, ±15.4
**SF**	57.8, ±32.7	13.6, ±6.0	41.9, ±21.1	9.7, ±3.6
**SI**	1278.0, ±723.9	71.8, ±65.0	982.8, ±364.2	46.3, ±28.1
**SS**	700.6, ±860.7	57.4, ±49.5	407.5, ±454	40.5, ±25.0
**STN**	124.4, ±78.1	20.0, ±11.8	116.4, ±96.7	13.2, ±5.1

**SUM**	475.2, ±235.5	211.4, ±145.1	370.8, ±110.8	144.5, ±54.0
**TEa**	205.6, ±122.0	78.6, ±58.0	136.7, ±45.9	49.3, ±29.6
**TRS**	27.8, ±18.0	20.4, ±19.1	24.1, ±16.7	14.6, ±11.3
**TT**	929.4, ±512.0	214.6, ±194.3	802.0, ±531.2	131.0, ±95.0
**TU**	531.4, ±445.1	60.8, ±36.4	353.3, ±263.6	41.7, ±18.4
**VIS**	68.4, ±49.9	5.0, ±4.1	42.7, ±26.3	3.6, ±3.7
**VISC**	138.6, ±109.1	22.0, ±21.0	86.0, ±49.0	14.3, ±9.3
**VLPO**	57.4, ±30.1	17.8, ±15.3	40.3, ±11.4	11.3, ±9.3
**VM**	821.2, ±745.3	84.8, ±34.6	523.4, ±351.8	63.8, ±20.3
**VMH**	1462.6, ±799.9	187.4, ±149.2	1004.7, ±629.3	137.7, ±133.4
**VP**	163.2, ±158.4	28.0, ±12.4	110.7, ±75.8	21.4, ±5.6
**ZI**	1829.0, ±912.2	263.0, ±107.7	1374.4, ±334.2	194.9, ±34.7

For each structure, the absolute number of neurons projecting to LHA is reported as mean ± SD and independently for ipsi- and contralateral hemispheres. Normalization for a total of 50k projecting neurons is also provided for ipsilateral and contralateral counts.
