# Supplementary material for: Disruption of orbitofrontal-hypothalamic projections in a murine ALS model and in human patients
Source: Transl Neurodegener. 2021 May 31;10:17. doi: 10.1186/s40035-021-00241-6 (PMC8168014; doi:10.1186/s40035-021-00241-6)
Supplement: Supplementary file 2 — Additional file 2: Supplemental file 2. Fig. S1. Altered cortico-hypothalamic projection pattern in mSOD1 mice at P95, absolute ipsilateral counts. Fig. S2. Altered cortico-hypothalamic projection pattern in mSOD1 mice at P95, contralateral hemisphere. Fig. S3. 50k normalized contralateral projection in P95 mSOD1 animals and MOp/SS atrophy in P95 mSOD mice. Fig. S4. Altered cortico-hypothalamic projection pattern in manually registered mSOD1 mice at P95, ipsilateral. Fig. S5. Altered projection pattern in manually annotated mSOD1 mice at P95, contralateral. Fig. S6. Unaltered cortico-hypothalamic projection pattern in mSOD1 mice at P25, contralateral hemisphere. Fig. S7. Altered cortico-hypothalamic projection pattern in kiFUS mice at P255, absolute counts ipsilateral. Fig. S8. Altered cortico-hypothalamic projection pattern in kiFUS mice at P255, contralateral hemisphere. [file 40035_2021_241_MOESM2_ESM.pdf]

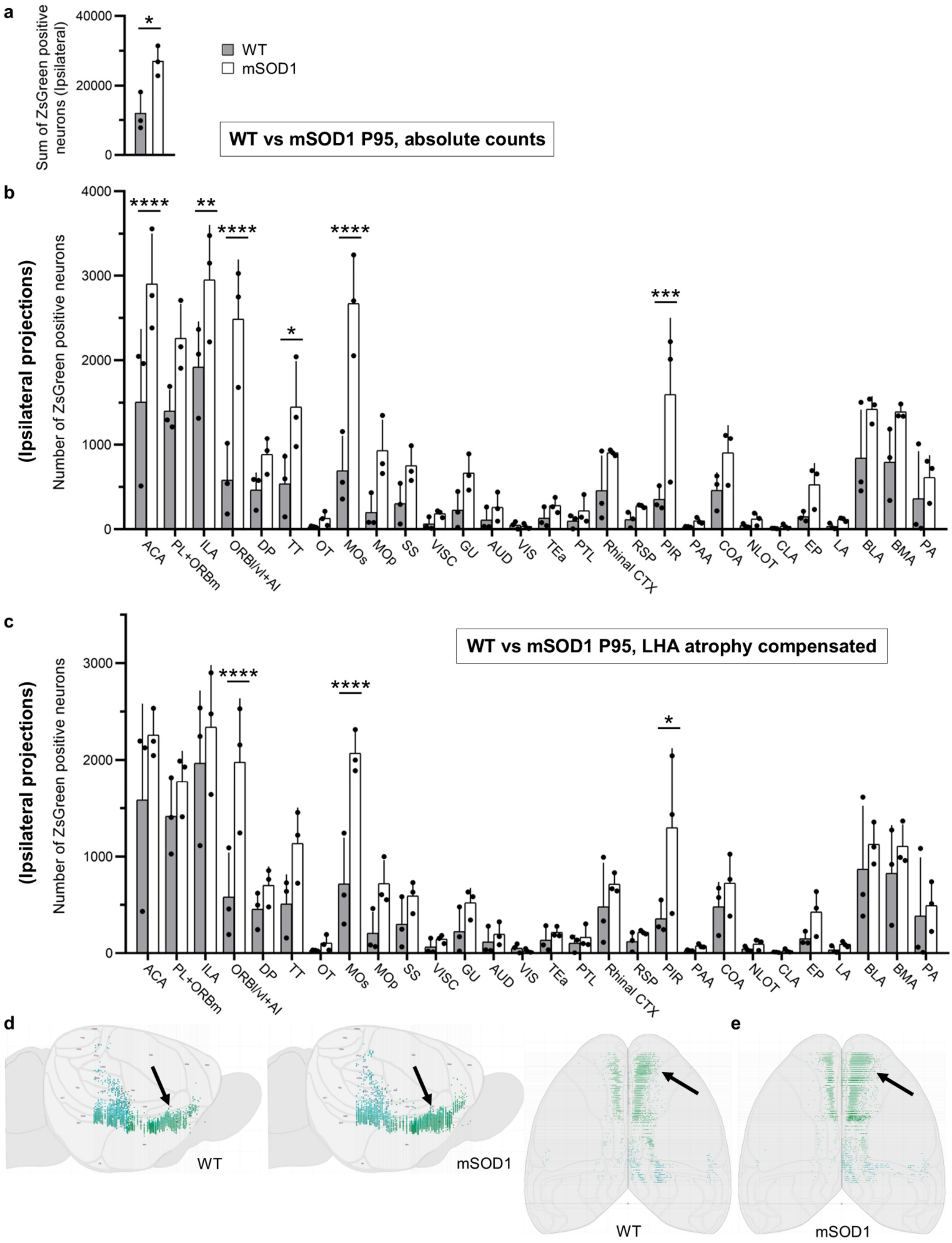
a
40000
20000
0
*
Sum of ZsGreen positive neurons (Ipsilateral)
WT
mSOD1
WT vs mSOD1 P95, absolute counts
b
4000
3000
2000
1000
0
(Ipsilateral projections)
Number of ZsGreen positive neurons
****
**
****
*
****
***
ACA
PL+ORBm
ILA
ORBl/vl+AI
DP
TT
OT
MOs
MOp
SS
VISC
GU
AUD
VIS
TEa
PTL
Rhinal CTX
RSP
PIR
PAA
COA
NLOT
CLA
EP
LA
BLA
BMA
PA
c
WT vs mSOD1 P95, LHA atrophy compensated
3000
2000
1000
0
(Ipsilateral projections)
Number of ZsGreen positive neurons
****
****
*
ACA
PL+ORBm
ILA
ORBl/vl+AI
DP
TT
OT
MOs
MOp
SS
VISC
GU
AUD
VIS
TEa
PTL
Rhinal CTX
RSP
PIR
PAA
COA
NLOT
CLA
EP
LA
BLA
BMA
PA
d
WT
mSOD1
e
WT
mSOD1

Fig. S1 Altered cortico-hypothalamic projection pattern in mSOD1 mice at P95, absolute ipsilateral counts. **a** Sum of neurons projecting from selected 28 areas. Projections to the LHA from the ipsilateral hemisphere are significantly increased in mSOD mice ($P = 0.02$; $N = 3$). **b** Absolute number of neurons projecting to LHA from 28 brain structures in WT and mSOD1. A significant increase in projections from ACA ($P < 0.0001$), ILA ($P = 0.009$), ORBl/vl+AI ($P < 0.0001$), Tenia Tecta (TT; $P = 0.0365$), secondary Motor Cortex (MOs, $P < 0.0001$) and Piriform cortex (PIR; $P = 0.0005$). **c** LHA atrophy compensated projections to LHA from 28 brain structures in WT and mSOD1. A significant increase in projections from ORBl/vl+AI ($P<0.0001$), MOs ($P<0.0001$) and PIR ($P=0.0231$) is detected. **d** Representative *WholeBrain* ipsilateral side view reconstructions of neurons projecting to LHA in WT and mSOD1 mice. Expansion of projections from MOs are visible (arrows). **e** Representative *WholeBrain* cortical top view reconstructions of neurons projecting to LHA in WT and mSOD1 mice. Increased projections from MOs are visible (arrows). Bars show mean ± SD. $^*P < 0.05$, $^{**}P < 0.01$, $^{***}P < 0.001$, $^{****}P < 0.0001$.

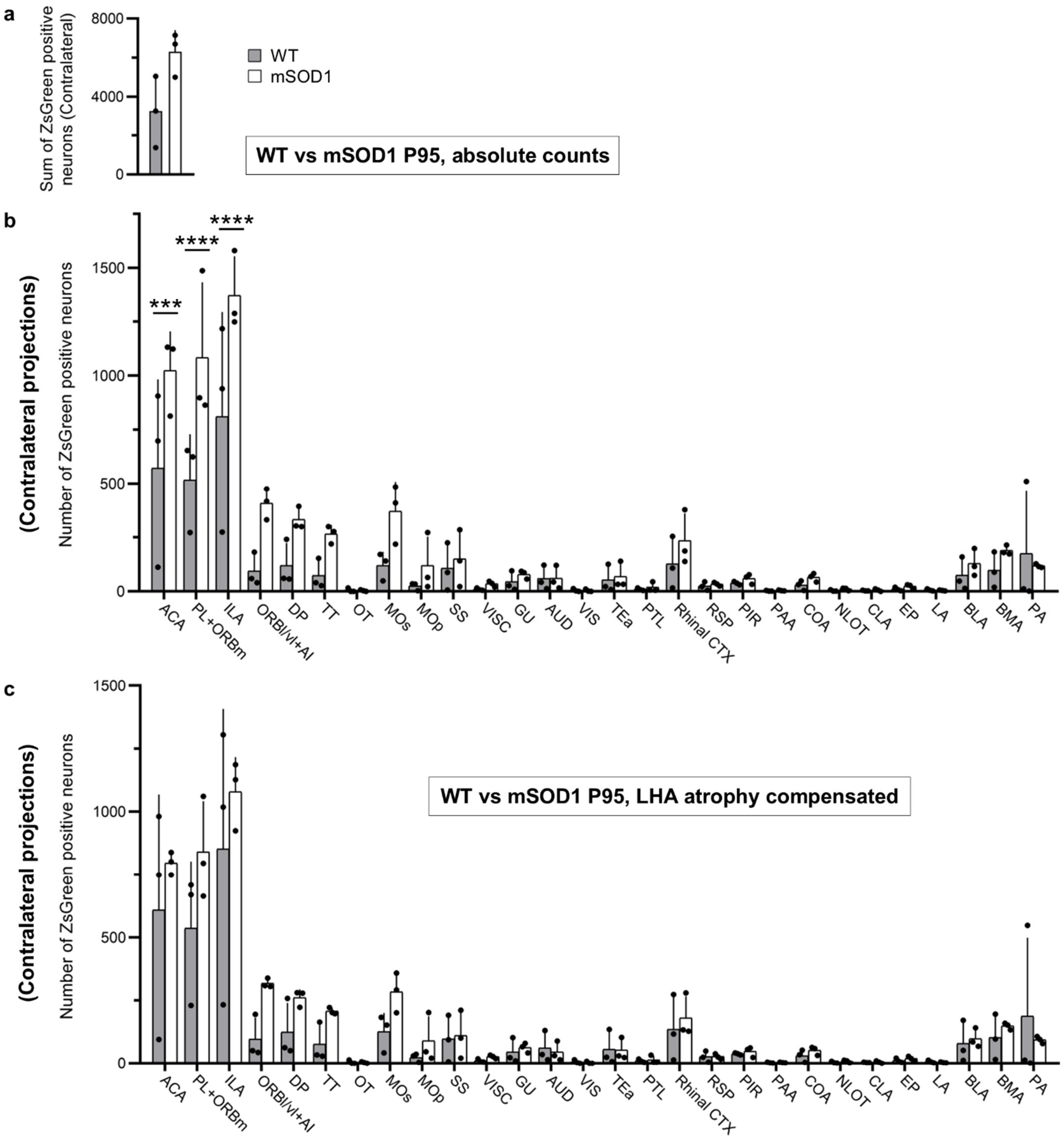
a
Sum of ZsGreen positive neurons (Contralateral)
8000
4000
0
WT
mSOD1
WT vs mSOD1 P95, absolute counts
b
(Contralateral projections)
Number of ZsGreen positive neurons
***
****
****
1500
1000
500
0
ACA
PL+ORBm
ILA
ORBl/vl+AI
DP
TT
OT
MOs
MOp
SS
VISC
GU
AUD
VIS
TEa
PTL
Rhinal CTX
RSP
PIR
PAA
COA
NLOT
CLA
EP
LA
BLA
BMA
PA
c
(Contralateral projections)
Number of ZsGreen positive neurons
WT vs mSOD1 P95, LHA atrophy compensated
1500
1000
500
0
ACA
PL+ORBm
ILA
ORBl/vl+AI
DP
TT
OT
MOs
MOp
SS
VISC
GU
AUD
VIS
TEa
PTL
Rhinal CTX
RSP
PIR
PAA
COA
NLOT
CLA
EP
LA
BLA
BMA
PA

Fig. S2 Altered cortico-hypothalamic projection pattern in mSOD1 mice at P95, contralateral hemisphere. **a** Sum of neurons projecting from selected 28 areas. No difference is detected in the number of neurons projecting to LHA in WT and mSOD1 from the contralateral hemisphere (*n*=3). **b** Absolute number of neurons projecting to LHA from 28 brain structures in WT and mSOD1. A significant increase in projections from ACA (*P*=0.0008), PL+ORBm and ILA (both *P*<0.0001) is detected. **c** LHA atrophy compensated projections to LHA from 28 brain structures in WT and mSOD1. No difference is detected in the number of neurons projecting to LHA from 28 brain structures in WT and mSOD1. Bars show mean ± SD. ***P < 0.001, ****P< 0.0001.

a

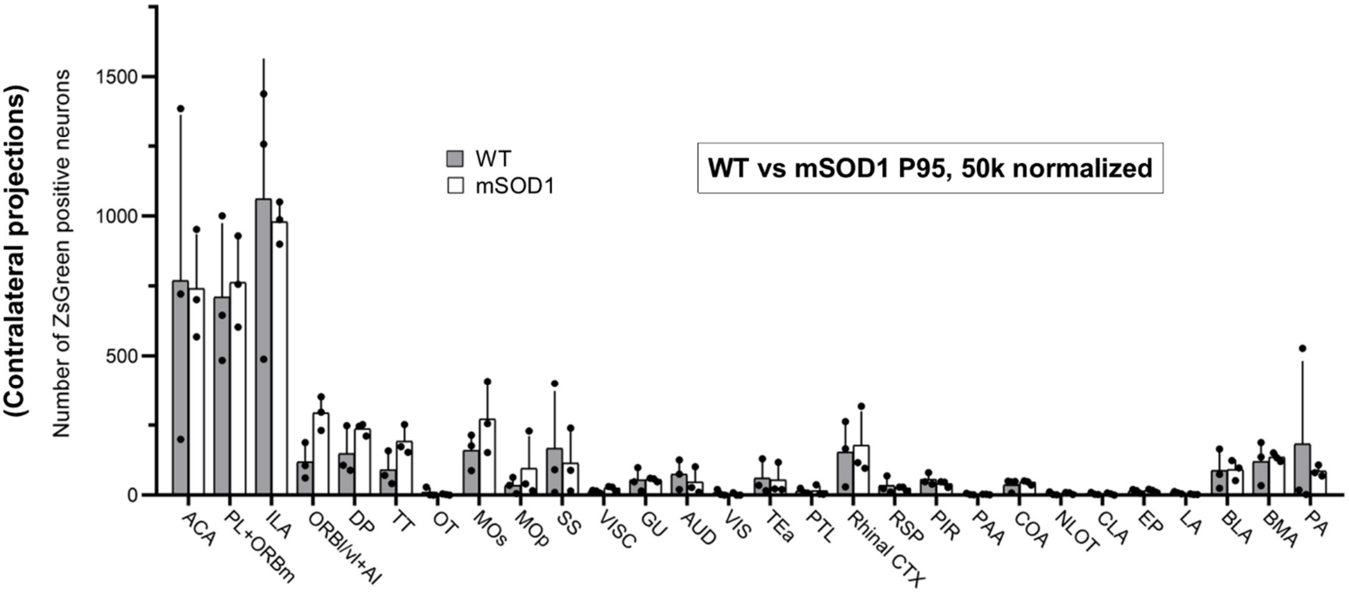

(Contralateral projections)
Number of ZsGreen positive neurons
1500
1000
500
0
WT
mSOD1
WT vs mSOD1 P95, 50k normalized
ACA
PL+ORBm
ILA
ORBl/vl+AI
DP
TT
OT
MOs
MOp
SS
VISC
GU
AUD
VIS
TEa
PTL
Rhinal CTX
RSP
PIR
PAA
COA
NLOT
CLA
EP
LA
BLA
BMA
PA

b

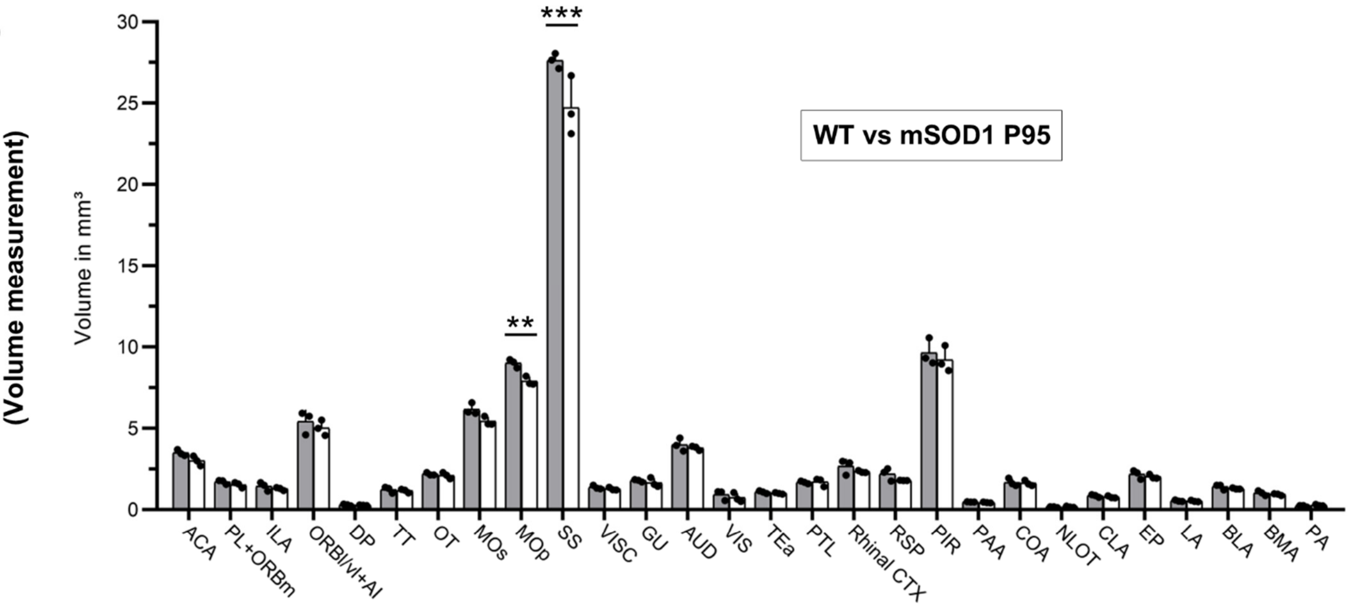

(Volume measurement)
Volume in mm³
30
25
20
15
10
5
0
WT vs mSOD1 P95
**
***
ACA
PL+ORBm
ILA
ORBl/vl+AI
DP
TT
OT
MOs
MOp
SS
VISC
GU
AUD
VIS
TEa
PTL
Rhinal CTX
RSP
PIR
PAA
COA
NLOT
CLA
EP
LA
BLA
BMA
PA

Fig. S3 50k normalized contralateral projection in P95 mSOD1 animals and MOp/SS atrophy in P95 mSOD mice. **a** No difference is detected in the 50k normalized number of neurons projecting to LHA from 28 brain structures in WT and mSOD1. **b** Volumetric comparison of 28 areas in WT and mSOD. Significant atrophy was detected in MOp (P=0.0080) and SS (P<0.0001). Bars show mean ± SD. ***P* < 0.01, ****P* < 0.001.

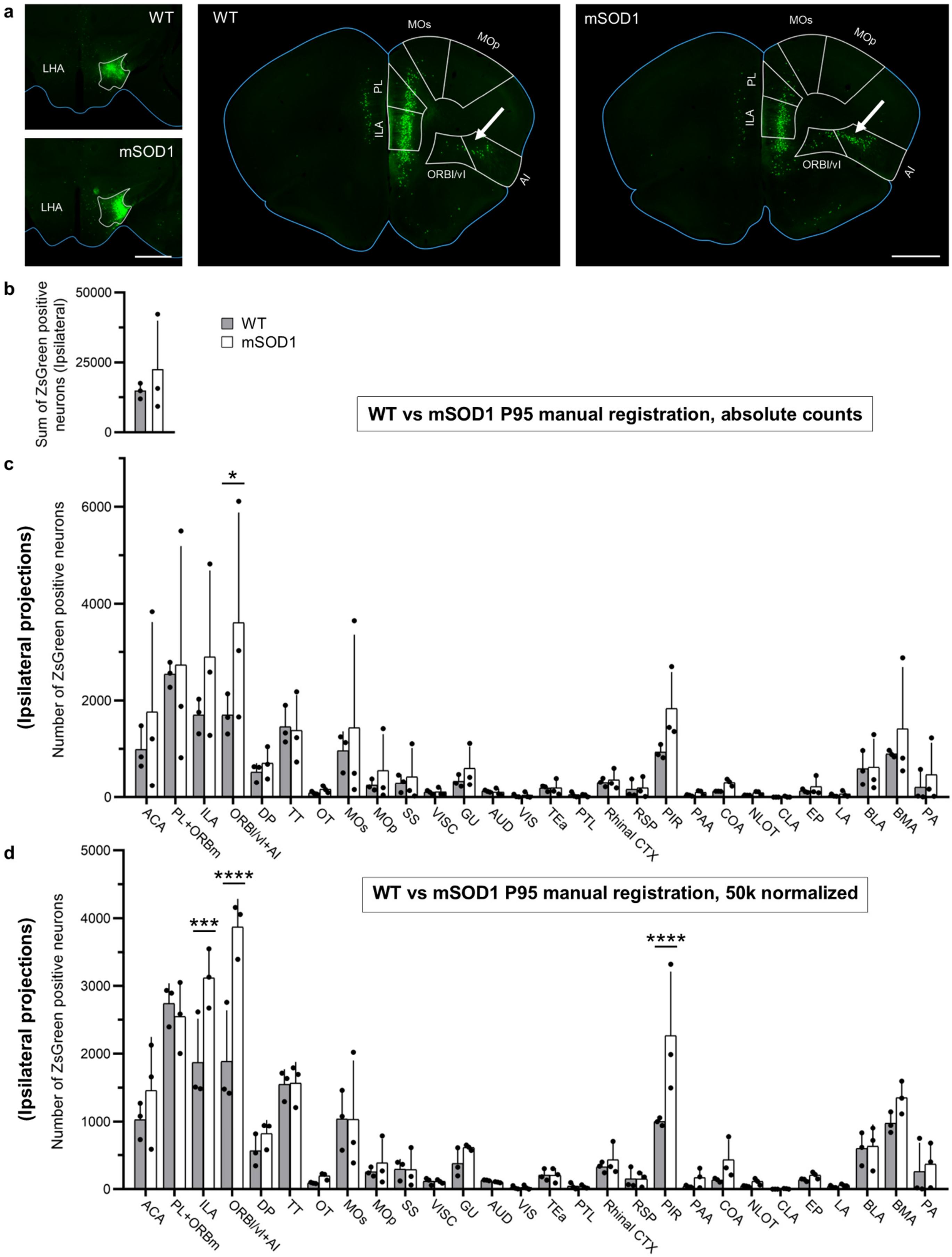
a
WT
LHA
mSOD1
LHA
WT
MOs
MOp
PL
ILA
ORBl/vl
AI
mSOD1
MOs
MOp
PL
ILA
ORBl/vl
AI
b
Sum of ZsGreen positive neurons (Ipsilateral)
50000
25000
0
WT
mSOD1
WT vs mSOD1 P95 manual registration, absolute counts
c
(Ipsilateral projections)
Number of ZsGreen positive neurons
6000
4000
2000
0
*
ACA
PL+ORBm
ILA
ORBl/vl+AI
DP
TT
OT
MOs
MOp
SS
VISC
GU
AUD
VIS
TEa
PTL
Rhinal CTX
RSP
PIR
PAA
COA
NLOT
CLA
EP
LA
BLA
BMA
PA
d
WT vs mSOD1 P95 manual registration, 50k normalized
(Ipsilateral projections)
Number of ZsGreen positive neurons
5000
4000
3000
2000
1000
0
***
****
****
ACA
PL+ORBm
ILA
ORBl/vl+AI
DP
TT
OT
MOs
MOp
SS
VISC
GU
AUD
VIS
TEa
PTL
Rhinal CTX
RSP
PIR
PAA
COA
NLOT
CLA
EP
LA
BLA
BMA
PA

Fig. S4 Altered cortico-hypothalamic projection pattern in manual registered mSOD1 mice at P95, ipsilateral. **a** Left: representative injection sites in LHA for WT and mSOD1 mice. White outlines represent LHA boundaries. Middle and right: Representative frontal brain sections of WT and mSOD1 mice depicting projections from ORBl/vl+AI (significantly increased, arrow), PL+ORBm, ILA, ACA, MOs and MOp to LHA. **b** Sum of neurons projecting from selected 28 areas. No difference is detected in the number of neurons projecting to LHA in WT and mSOD1 from the ipsilateral hemisphere (*n*=3). **c** Absolute number of neurons projecting to LHA from 28 brain structures in WT and mSOD1. A significant increase in projections from ORBl/vl+AI (*P*=0.0352) is detected. **d** 50k normalized number of neurons projecting to LHA from 28 brain structures in WT and mSOD1. A significant increase in projections from ILA (*P*=0.0001), ORBl/vl+AI (*P*<0.0001) and PIR (*P*<0.0001) is detected. Bars show mean ± SD. Scale bars 1 mm. **P* < 0.05, ***P* < 0.01, ****P* < 0.001, *****P*< 0.0001.

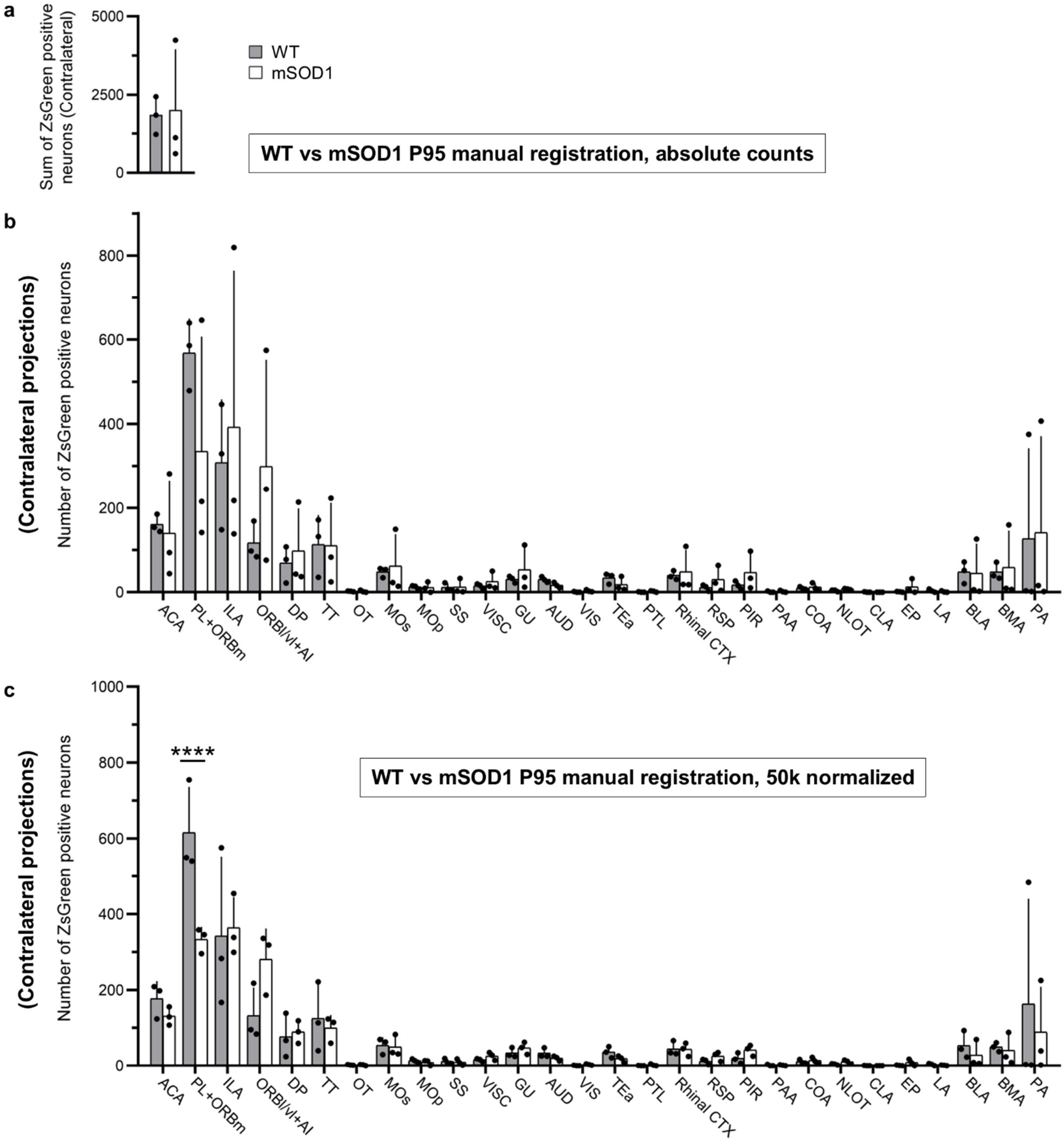
a
b
c
Sum of ZsGreen positive neurons (Contralateral)
WT
mSOD1
WT vs mSOD1 P95 manual registration, absolute counts
(Contralateral projections)
Number of ZsGreen positive neurons
WT vs mSOD1 P95 manual registration, 50k normalized
****
ACA
PL+ORBm
ILA
ORBl/vl+AI
DP
TT
OT
MOs
MOp
SS
VISC
GU
AUD
VIS
TEa
PTL
Rhinal CTX
RSP
PIR
PAA
COA
NLOT
CLA
EP
LA
BLA
BMA
PA

Fig. S5 Altered projection pattern in manually annotated mSOD1 mice at P95, contralateral. **a** Sum of neurons projecting from selected 28 areas. No difference is detected in the number of neurons projecting to LHA in WT and mSOD1 from the contralateral hemisphere (*n*=3). **b** No difference is detected in the absolute number of neurons projecting to LHA from 28 brain structures in WT and mSOD1. **c** 50k normalized number of neurons projecting to LHA from 28 brain structures in WT and mSOD1. A significant increase in projections from PL+ORBm (P<0.0001) is detected. Bars show mean ± SD. ****P< 0.0001.

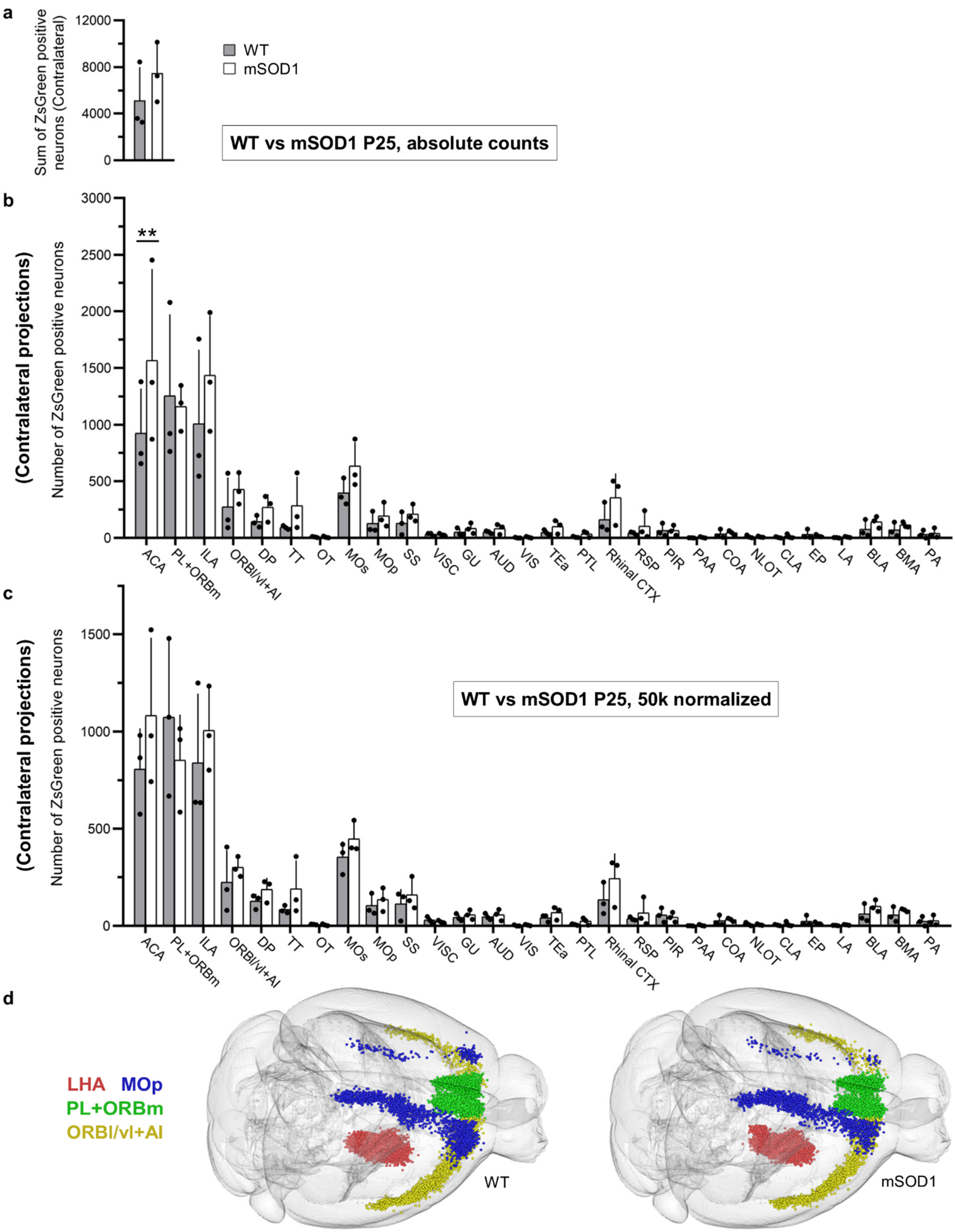
a
Sum of ZsGreen positive neurons (Contralateral)
12000
8000
4000
0
WT
mSOD1
WT vs mSOD1 P25, absolute counts
b
(Contralateral projections)
Number of ZsGreen positive neurons
3000
2500
2000
1500
1000
500
0
**
ACA
PL+ORBm
ILA
ORBl/vl+AI
DP
TT
OT
MOs
MOp
SS
VISC
GU
AUD
VIS
TEa
PTL
Rhinal CTX
RSP
PIR
PAA
COA
NLOT
CLA
EP
LA
BLA
BMA
PA
c
(Contralateral projections)
Number of ZsGreen positive neurons
1500
1000
500
0
WT vs mSOD1 P25, 50k normalized
ACA
PL+ORBm
ILA
ORBl/vl+AI
DP
TT
OT
MOs
MOp
SS
VISC
GU
AUD
VIS
TEa
PTL
Rhinal CTX
RSP
PIR
PAA
COA
NLOT
CLA
EP
LA
BLA
BMA
PA
d
LHA
MOp
PL+ORBm
ORBl/vl+AI
WT
mSOD1

Fig. S6 Unaltered cortico-hypothalamic projection pattern in mSOD1 mice at P25, contralateral hemisphere. **a** Sum of neurons projecting from selected 28 areas. No difference is detected in the number of neurons projecting to LHA in WT and mSOD1 from the contralateral hemisphere (*n*=3). **b** Absolute number of neurons projecting to LHA from 28 brain structures in WT and mSOD1. A significant increase in projections from ACA (*P*=0.0078) is detected. **c** 50k normalized number of neurons projecting to LHA from 28 brain structures in WT and mSOD1. No difference is detected in the number of neurons projecting to LHA from 28 brain structures in WT and mSOD1. **d** Representative *WholeBrain* mesoscale reconstructions of neurons projecting to LHA in WT and mSOD1 mice show a similar pattern of projections in MOs (blue), ORBl/vl+AI (yellow) and PL+AI (green). Bars show mean ± SD. $^{**}P < 0.01$.

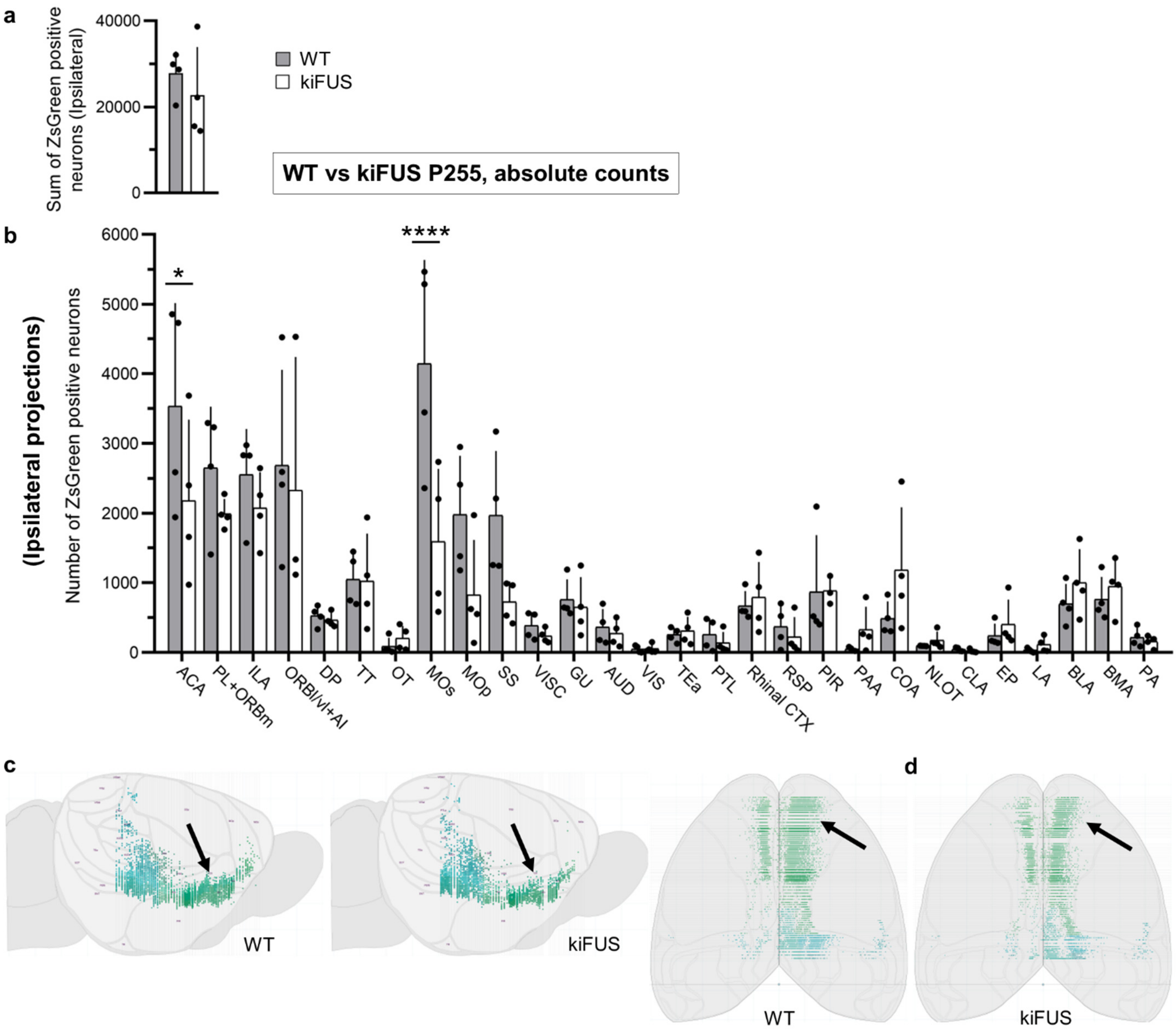
a
Sum of ZsGreen positive neurons (Ipsilateral)
40000
20000
0
WT
kiFUS
WT vs kiFUS P255, absolute counts
b
(Ipsilateral projections)
Number of ZsGreen positive neurons
6000
5000
4000
3000
2000
1000
0
*
****
ACA
PL+ORBm
ILA
ORBl/vl+AI
DP
TT
OT
MOs
MOp
SS
VISC
GU
AUD
VIS
TEa
PTL
Rhinal CTX
RSP
PIR
PAA
COA
NLOT
CLA
EP
LA
BLA
BMA
PA
c
WT
kiFUS
d
WT
kiFUS

Fig. S7 Altered cortico-hypothalamic projection pattern in kiFUS mice at P255, absolute counts ipsilateral. **a** Sum of neurons projecting from selected 28 areas. No difference is detected in the number of neurons projecting to LHA in WT and kiFUS from the ipsilateral hemisphere (*n*=4). **b** Absolute number of neurons projecting to LHA from 28 brain structures in WT and mSOD1. A significant increase in projections from ACA (P=0.0385) and MOs (P<0.0001) is detected. **c** Representative *WholeBrain* ipsilateral side view reconstructions of neurons projecting to LHA in WT and mSOD1 mice. Decrease of projections from MOs are visible (arrows). **d** Representative *WholeBrain* cortical top view reconstructions of neurons projecting to LHA in WT and mSOD1 mice. Decrease of projections from MOs are visible (arrows). Bars show mean ± SD. *P < 0.05, ****P< 0.0001.

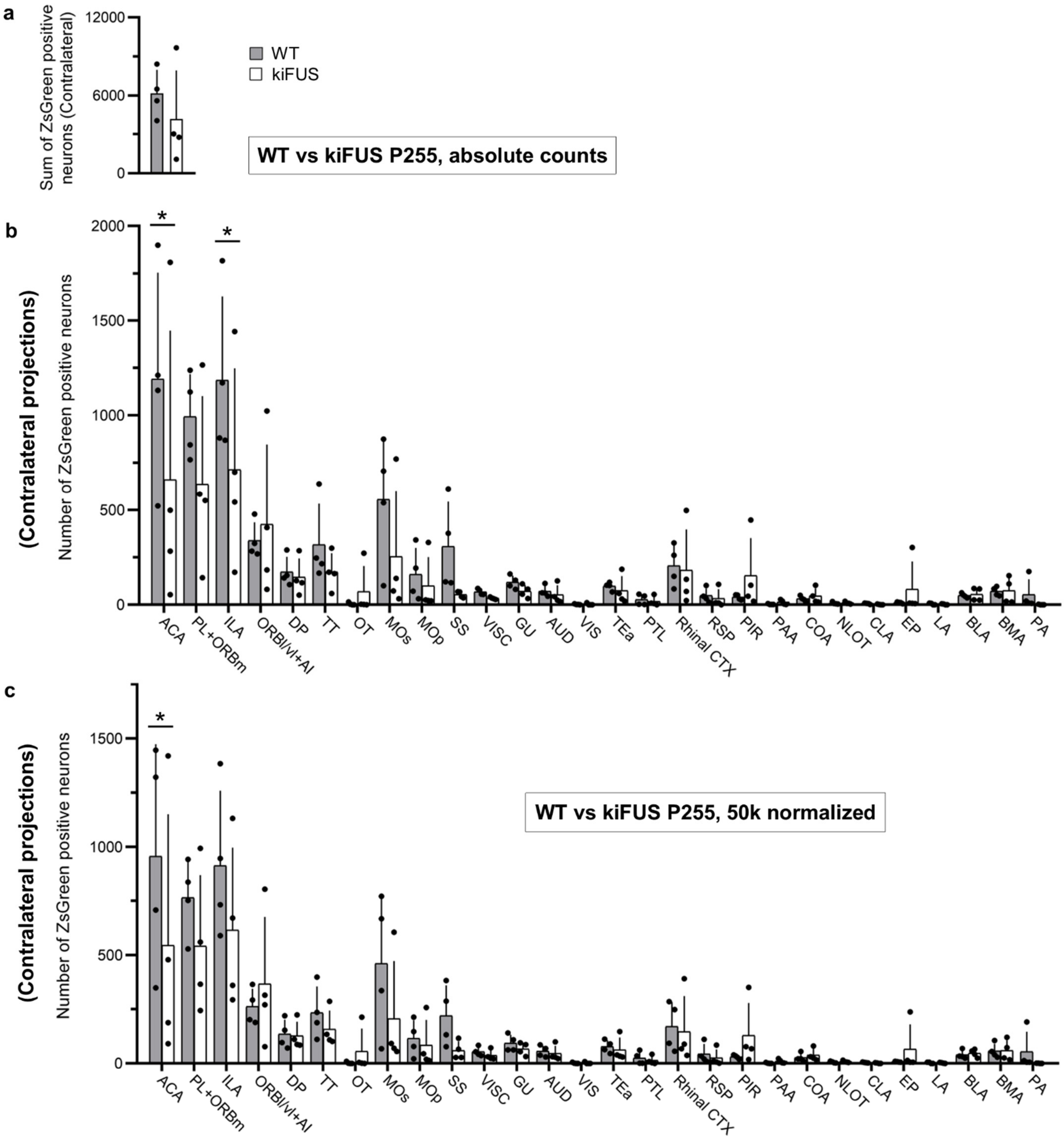
a
b
c
WT
kiFUS
WT vs kiFUS P255, absolute counts
WT vs kiFUS P255, 50k normalized
Sum of ZsGreen positive neurons (Contralateral)
(Contralateral projections)
Number of ZsGreen positive neurons
12000
6000
0
2000
1500
1000
500
*
ACA
PL+ORBm
ILA
ORBl/vl+AI
DP
TT
OT
MOs
MOp
SS
VISC
GU
AUD
VIS
TEa
PTL
Rhinal CTX
RSP
PIR
PAA
COA
NLOT
CLA
EP
LA
BLA
BMA
PA

Fig. S8 Altered cortico-hypothalamic projection pattern in kiFUS mice at P255, contralateral hemisphere. **a** Sum of neurons projecting from selected 28 areas. No difference is detected in the number of neurons projecting to LHA in WT and kiFUS from the contralateral hemisphere (*n*=3). **b** Absolute number of neurons projecting to LHA from 28 brain structures in WT and mSOD1. A significant increase in projections from ACA (*P*=0.0121) and ILA (*P*=0.0474) is detected. **c** 50k normalized number of neurons projecting to LHA from 28 brain structures in WT and kiFUS. A significant decrease in projections from ACA (*P*=0.0137) is detected. Bars show mean ± SD. *$P < 0.05$.
